# Translational AI in whole-slide image cancer histopathology: state of the art and regulatory-approved solutions

**DOI:** 10.3389/fdgth.2026.1863382

**Published:** 2026-07-09

**Authors:** Richard Oliver Matzko, Burak Kucukgoz, Pawel Gertner, Christopher Carey, Chris M. Bacon, Tong Xin

**Affiliations:** 1 Translational and Clinical Research Institute, Newcastle University, Newcastle upon Tyne, United Kingdom; 2 Department of Computing, Newcastle University, Newcastle upon Tyne, United Kingdom; 3 Dept Cellular Pathology, Newcastle upon Tyne Hospitals NHS Foundation Trust, Newcastle upon Tyne, United Kingdom; 4 Wolfson Childhood Cancer Research Centre, Translational and Clinical Research Institute, Newcastle University, Newcastle upon Tyne, United Kingdom

**Keywords:** artificial intelligence, biomarker assessment, computational pathology, digital histopathology, machine learning, regulatory approval, whole-slide imaging

## Abstract

With an emphasis on applied evidence, we present an in-depth evaluation of artificial intelligence (AI) in cancer histopathology through the lens of United States Food and Drug Administration (FDA)-approved and European Conformity-marked whole-slide image *In Vitro* Diagnostic Medical Devices. Having identified only four existing FDA-approved whole-slide image cancer solutions for a narrow range of applications, we conclude that AI in digital histopathology remains in an emerging state. Best practices were identified by examining development and validation evidence across market-approved solutions. Findings were contrasted with state-of-the-art research-only AI histopathology pipelines. Insights were drawn regarding applications, learning modalities, processing strategies, statistical methods, and validation approaches. Regulatory guidelines were evaluated from FDA and UK Government documentation as well as academic literature, with patient safety highlighted as a central concern. Approved products were observed to integrate efficiently into existing clinical decision-making frameworks, with future potential to enhance the use of pathologist consensus in AI applications. Biomarker assays may be coupled specifically to emerging therapies, but challenges remain for direct clinical adoption outside the research-only sphere. Although hurdles remain in validating agentic and generative AI for medicine, further adoption of state-of-the-art algorithmic frameworks—including transformer architectures and multimodal approaches—is anticipated. As pan-cancer systems emerge, computational modules and engineering principles may also be retargeted to underrepresented use cases. Consequently, this review provides a forward-looking framework for translational, market-relevant histopathology AI.

## 1 Introduction

We examine digital histopathology artificial intelligence (AI) for cancer, centered on whole-slide images (WSIs) as the primary modality. Our novel emphasis on regulation and market certification can assist researchers in bridging quality control and translational gaps. AI tools are particularly applicable in this role, since histopathology remains the gold standard for diagnosis and classification in cancers such as prostate and breast cancer (1). For example, Gleason grading is the most significant predictor of prostate cancer prognosis, with metastasis a key factor in determining management (2). This paper aims to support next-generation translational AI development by promoting understanding of regulatory and commercial deployment, validation strategies and metrics, statistical methods, and the WSI cancer AI ecosystem. We contextualize these insights with selected research-only state-of-the-art applications.

### 1.1 Artificial intelligence background

AI and Machine Learning (ML) provide objective-driven optimization, enabling the resolution of otherwise intractable problems. Deep Learning (DL) utilizes artificial neural networks (ANNs) for encoding and decoding with intermediary “latent” representations. AI research and development was recognized with the 2024 Nobel Prize (3) for development of ANNs for ML and AlphaFold for protein structure prediction.

AI-assisted decision-making is often referred to as “augmented intelligence” (4) or “adjunctive intelligence” (5), although clinical workflow enhancement, rather than pathologist replacement, can mitigate deskilling or decision-making biases (6). Historically, AI has demonstrated superhuman task-specific decision-making, perhaps associated with its consistency and scalability (7). Today, AI can reason, converse, autonomously research, and code through proprietary Large Language Models (LLMs) and multimodal models, such as OpenAI's ChatGPT, Microsoft's Copilot, Google's Gemini, and xAI's Grok. LLMs have been made publicly available through platforms including Hugging Face, which rated LLMs across capabilities (8).

LLMs have been proposed for enhancing and structuring pathology reports and improving patient communication (9). For example, in Nature research, Qwen2-7B-Instruct was used to remove sensitive information, with GPT4o-mini used to compare histopathology specimen reports (10). Such agentic and generative AI systems are capable of novel outputs and tend to be categorized within high-autonomy quadrants, with associated regulatory challenges and high-risk classification (11). Agentic AI responses have even been linked to patient confusion or harm (9). The AI Playbook for the UK Government warned that “LLMs are not domain experts” and “not a substitute for professional advice,” although this applies only where “precise and contextually relevant information is essential” (12). The authorized systems we identified for histopathology occupy constrained niches, representing narrower, non-autonomous, non-adaptive, and human-in-the-loop solutions. Self-learning adaptive AI is another regulatory concern (13), and the UK government emphasized that full automation of high-risk or high-impact decisions should be avoided for ethical, legal, and social reasons (12). Ironically, regulators pursue automation in source code review (13).

Software breakthroughs have been necessary, notably the attention mechanism (14, 15) which is critical to transformer architecture and enables algorithms to handle long-range informational dependencies. Transformer architecture dispensed with recurrence and convolutions to rely on multi-headed self-attention and was developed collaboratively by Google Brain and Google Research. Because this seminal work was evaluated on translation sentence pairs, the architecture naturally extended to user-assistant dialog systems. Efficient, long-sequence, high-throughput alternatives such as Mamba have been proposed (16), which excludes attention and couples a structured state space model with the multilayer perceptron block from transformers, albeit with limitations when tasked on dense information. The canonical equation (14, 17) for attention, $\mathrm{Attention}\;(Q,\;K,\;V)=\mathrm{softmax}\left(QK^{T}/\sqrt{d_{\mathrm{keys}}}\right)V$, is given by relating Query (*Q*), Key (*K*), and Value (*V*), and Key dimensions ($d_{\mathrm{keys}}$). Convolutional neural networks (CNNs) were considered the “de facto standard method” in WSI pathology research (18, 19). However, transformer-based architectures have emerged as state of the art (20), including vision-language models trained on vast WSI datasets and text–image pairs.

### 1.2 AI in WSI cancer histopathology

Digital histopathology utilizes digitized histopathological slides (18), involving the scanning, visualization, analysis, transfer, and storage of WSIs (21). Digitized slides can reach several gigabytes in size to maintain quality. Scanners undergo market approval by the US Food and Drug Administration (FDA), typically supporting ×20 and ×40 magnifications, and can accommodate slide volumes into the hundreds. Image management systems (IMS) may be vendor-specific or open platform, with variable FDA approval restrictions. Open IMS solutions include Viewr+, FlexLIS, Concentriq AP-Dx, and Sectra, while vendor-specific solutions are provided by companies such as Phillips, Leica, and Roche.

DL in WSI cancer histopathology can grade (22), subtype, diagnose, prognosticate, stratify prognostically (23), evaluate clinical and therapeutic responses (24, 25), perform object counting, score immunohistochemical stains, detect lesions, localize precancerous lesions (21), evaluate (4), and discover (26) biomarkers, as well as assess molecular aberrations (19, 20, 27). These aberrations are reflected as quantifiable microscopic patterns with predictive utility (20), for example, receptors, Ki-67, Programmed Death-Ligand 1 (PD-L1) protein markers, and Homologous Recombination Deficiency and Microsatellite Instability (MSI) biomarker states. AI medical devices can assist with triaging and priority ranking (24, 28, 29), providing second opinions (24), and supporting cancer treatment selection (23, 30). AI may also be used for stain (27) and slide (31) quality control.

Prompt cancer diagnosis can increase treatment options and long-term survival, while enabling less-invasive interventions (32). Prognosis and survival predictions are “crucial” for patient counseling and treatment decisions in lymphoma, although data acquisition can be challenging due to the need for long-term follow-up (33). AI may enhance personalized medicine (6), including avoidance of over- and under-treatment (34). For example, in colorectal cancer stages II and III, many patients have been cured by surgery alone, with the majority of patients at risk from side effects and financial costs of routine adjuvant chemotherapy (25% recurrence) (35). Nevertheless, treatment options clearly require careful consideration, since adjuvant therapy increased absolute survival by up to 10%–22% in stage III.

Biomarkers can be discerned from Hematoxylin and Eosin (H&E) stained slide characteristics or enhanced by molecular treatment such as immunohistochemistry (IHC). Identification of actionable biomarkers elevates the potential of precision medicine (31), which can guide immunotherapy (36). IHC is used to assess protein overexpression, while *in situ* hybridization is used to assess gene amplification (37). This review inevitably emphasizes H&E as the most common stain (30) associated with lower costs compared with IHC (4).

**Figure 1:**
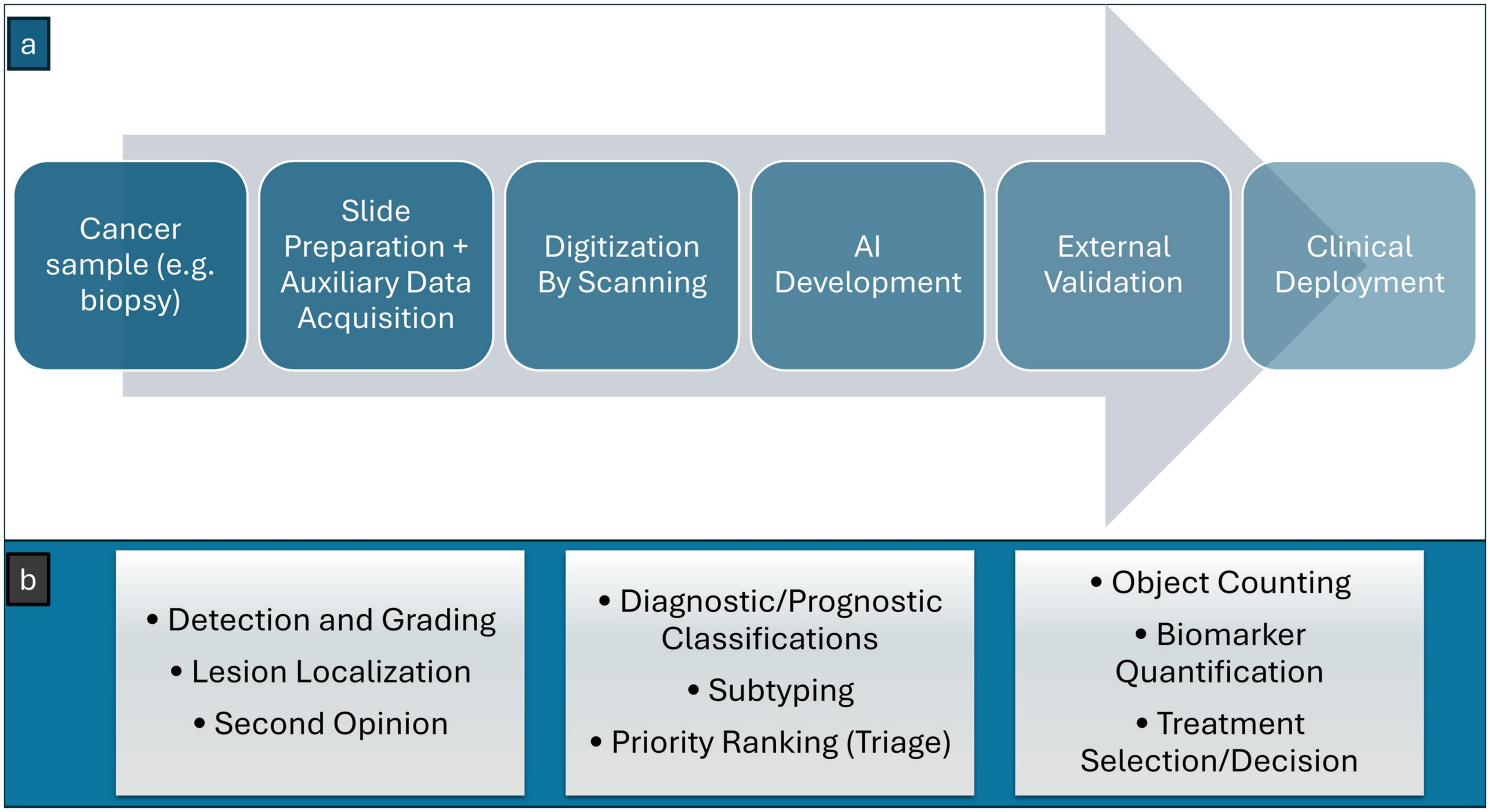
A generalized overview of the WSI AI development pipeline with applications. **(a)** The generalized pipeline from sample collection through to clinical AI deployment, a critical component of which is external validation. Note that auxiliary data may be desired for multimodal modeling. **(b)** Possible AI uses. Uses may overlap; for example, grading may result from biomarker quantification leading to a treatment decision.

With potential to reduce interobserver variability and detect small quantities of cancer (38), regulatory-approved AI can offer pathologists a safety net against missed cancers (39). Interobserver variability in PD-L1 assessment has been associated with inaccurate patient stratification in lung cancer (36). The CAMELYON17 Grand Challenge (CGC17) centered on WSIs, aiming to reduce pathologist workloads and diagnostic subjectivity (40). Thus, AI may increase efficiency, reduce costs, and enhance patient outcomes (30), including time saving for pathologists and patients (2, 31). This is notable in view of diagnostic pathologist (31, 38, 41) and radiologist (9) shortages, increasing global cancer incidence (1, 39, 41), precision medicine complexity (39), and growing pathologist workloads (41). Cost savings may incentivize AI adoption in hospitals, although costs remain ill-defined due to software (4) and training expenses (20). Figure 1 provides a general overview of the WSI AI development pipeline.

### 1.3 The regulatory environment

Regulatory perspectives establish market standards (28), although regulators must balance regulation with innovation (13, 42). The UK system allows sector-specific interpretation to foster innovation (43). Important regulatory bodies include the FDA and the European Conformité Européenne (CE) for *In Vitro* Diagnostic standards (CE-IVDR) (25). The UK regulates through the Medicines and Healthcare products Regulatory Agency (MHRA) (44), with the National Institute for Health and Care Excellence (NICE) providing recommendations and standards of appropriate treatment and care within NHS England and Wales (45). NICE also assesses AI (46).

In Japan, the Pharmaceuticals and Medical Devices Agency is involved in developing AI regulatory frameworks, while in China this role is fulfilled by the National Medical Products Administration (13). Within the European Economic Area, AI image analysis software is subject to *In Vitro* Diagnostic Medical Devices Regulation (IVDR), which has a tendency to classify them as higher risk (13). In the UK, IVDs may be marketed under the UKCA or CE mark. In Europe, the CE mark is required for AI product marketing, following rigorous clinical evaluations and validation (13). This review emphasizes European Union (EU), US, and UK approval pathways, summarized according to our findings in Table 1.

**Table 1:** Comparative overview of regulatory and certification mechanisms relevant to AI-enabled WSI histopathology systems across the USA, EU, and UK.

Jurisdiction/body	Main regulatory or certification mechanism	Risk classification/interpretive note
USA/FDA	FDA premarket pathways, including 510(k), *de novo*, Premarket Approval, and Humanitarian Device Exemption	Risk classes I–III. Several identified WSI AI tools are Class II. WSI systems for cancer applications tend to be classified as higher risk
EU/CE-IVDR	CE-IVD marking under IVDR	Risk-based classification. Image analysis IVD software tends toward higher-risk classification
UK/MHRA	UKCA or accepted CE routes, with MHRA registration where applicable	Risk-based classification according to intended purpose. MHRA registration does not itself imply approval, certification, or endorsement
Health institution/in-house exemptions	Clinical Laboratory Improvement Amendments context in the USA; MHRA Health Institution Exemption in the UK	Internal/in-house use is not conventional commercial market authorization and depends on jurisdiction, intended use, and availability of alternatives

### 1.4 The importance of validation and use case applicability

Generalizability of medical AI models is widely sought, such that AI effectively handles external cohort (23, 24, 41, 47) variability, requiring diverse (24, 30, 47) and representative (47) test datasets, with deployment strictly within validated environments. Generalization may span demographics, sample preparation, and data acquisition (48). Inclusivity (12) and representativeness of modeling across age, sex, race, and ethnicity (44) allow for ethical and performant usage, with subgroup analysis advised to ensure health equity (13). Disease severity, disease variables, and clinical site are other performance stratification concerns (28). A lack of prospective clinical trials compared with retrospective studies has been observed (4, 13), with randomized controlled studies recommended by the FDA (12).

Lesion detection may not be causal to improved prognoses (13). Associative modeling does not guarantee a causal link within a classifier (47), thus highlighting overfitting risks for unexpected, spurious biases. Spurious correlations are a risk if validation data contain the same confounding characteristics as the development data (28). It is critical to avoid data leakage (28) between training and validations sets (44), including experiments on public datasets using AI models trained on them (22). DL models have even performed “perfectly” on randomly generated target variables (47), highlighting the need for rigorous validation. AI-powered histopathology research has often been noted to contain errors in validation methodology (48), further justifying interest in regulatory-approved trajectories.

## 2 Research method

Four FDA-authorized AI WSI pathology solutions were identified by searching for “pathology” on the FDA Artificial Intelligence-Enabled Medical Devices database (49). Of these, one was cytological (50, 51). Several pathways to FDA approval exist, including 510(k), Premarket Approval (PMA), humanitarian device exemption, and the *de novo* pathway (52). Products designated under 510(k) were identified via Innolitics with the “Contains AI/ML” filter applied (50). The FDA Breakthrough Devices Program (BDP) only provides pre-approval designation (53, 54), although the BDP website lists devices that reached market authorization (53). The BDP included 46 pathological and 43 radiological devices, among other domains, that reached marketing authorization (53).

European device searches were unintuitive, with devices of interest identified through AI-guided web searches and article references. IVD approval has been criticized as non-transparent, with poor archiving of data acquisition, training, and validation methods (27). Consequently, European AI medical devices may benefit from improved archiving and search capabilities. The EU Database on Medical Devices (EUDAMED) is under development to offer searches for product certifications, approvals, and associated companies (30). On EUDAMED, search codes can be used to identify specific applications. Overall, the pool of identified CE-approved devices for WSI histopathology was larger than that of the FDA. At least nine CE-approved devices are discussed in this review. The MHRA Public Access Registration Database can also be used to search across registered companies, including by code (30, 55), although it provides only registration details and notes that this “does not represent … accreditation, certification, approval or endorsement.” ChatGPT 5.1 and 5.2 by OpenAI (56) were leveraged for search assistance.

Academic literature was sourced from ongoing academic investigations, internal discussions, company websites for validation studies and trials, and abstract relevance. All journal articles, reviews, editorials, conference papers, and preprints were available on and cross-checked through Scopus (57). Search criteria focused on medically approved AI devices for pathology, emphasizing WSI histopathology and regulatory AI governance. We contrast that emphasis with pure AI histopathology research and support it with statistical resources. For a summary of paper references, see Table 2. The investigation concluded in February 2026 prior to final consolidation.

**Table 2:** A catalogue of the resource types referenced in this review.

Source type	Count	Sources (count)	Role in review	Year range
Scopus: journal articles, reviews, editorials, and conference papers	49/119	Nature Portfolio (12), Lancet family (4), Pathological Society (2), JCO Precision Oncology (2), AAAI (2), Statistics in Medicine (2), other scholarly journals/venues (25)	AI validation and clinical trial studies; medical AI reviews of decision support, cancer pathology, public evidence, and regulation; historical AI context; foundation model and multiple instance learning algorithms and pipelines; algorithm benchmarking; transformer architecture; statistical analysis and metrics; prognostic modeling; slide annotation; learning mechanisms; within- and out-of-domain algorithm comparison; pan-cancer algorithms; non-computational clinical consensus context; multimodal foundation model examples; and spatial biology and modality-enhancement context	1996–2026
Webpages: AI regulation	20/119	FDA (8), MHRA (4), Innolitics (3), UK Government (2), US Congress (1), NICE (1), NHS (1)	Regulatory guidance, technical evidence, decision summaries, AI implementation context, device and company registration search and verification	2021–2026
Webpages: AI histopathology companies	14/119	Ibex (3), Paige (2), Lunit AI (2), Roche (1), DoMore! Diagnostics (1), Limbus AI Inc. (1), Modella AI (1), Owkin (1), Visiopharm (1), DeepPath (1)	Company primary sources for AI histopathology product descriptions and validation study links	2022–2026
Webpages: databases	10/119	CAMELYON17 (2), National Cancer Institute (2), EUDAMED (1), Romion Health AI register (1), Royal College of Radiologists (1), EBRAINS (1), Zenodo (1), Scopus (1)	AI histopathology competition benchmarking and evidence, EUDAMED search, cross-modality context from radiology AI registers and omics portals, histopathology data access, and literature search	2019–2026
Webpages: code repositories	6/119	Hugging Face (3), GitHub (3)	LLM leaderboard, self-supervised learning source code, pickle-file cybersecurity, hosted dataset access, and histopathology pipeline source code	2022–2026
Webpages: other	8/119	IBM (2), Nobel Prize (1), OpenAI (1), National Library of Medicine (1), scikit-learn (1), Bruker (1), Illumina (1)	AI state-of-the-art, fine-tuning, mixture of experts, NICE, metric definition, and spatial biology/modality enhancement contexts	2025–2026
Books	3/119	O'Reilly Media (1), St Martin's Press (1), StatPearls Publishing (1)	Medical, ML, and statistical context	2007–2025
Scopus: preprints	5/119	arXiv (5)	ML algorithms and annotation levels context; multimodal foundation model and vision-language model examples	2020–2025
Webpages: company embedded videos	2/119	YouTube (2)	DoMore Diagnostics explanations	2021
Scopus: secondary documents	1/119	*Journal of Economy and Technology* (1)	AI regulatory context	2026
Webpages: policy commentary	1/119	whitecase.com (1	AI regulatory context	2025

Digital histopathology-relevant topics covered included AI regulation and regulatory decisions, company descriptions, AI device and clinical trial validation and benchmarking, underlying statistical and ML mechanisms, learning strategies, foundation model architectures, histopathology ML pipelines, cybersecurity, source code and dataset access, literature and registry search resources, cross-modality context from radiology and omics portals, AI tool listings, prognostic modeling, slide annotation strategies, and non-computational clinical consensus context.

## 3 Regulation and certification

This section distills best-practice regulatory guidelines, categorized into (1) expert supervision, multidisciplinary collaboration, and documentation (Supplementary Section S1); (2) performance risks and monitoring; and (3) cybersecurity and data security.

In 2021, the Good Machine Learning Practice (44) guidelines provided 10 principles to foster growth, as determined by the FDA (13), Health Canada, and the MHRA (44). In 2025, the AI Playbook for the UK Government was released as a collaboration between government, public sector, academia, and industry (12), emphasizing 10 principles for government and public sector AI adoption. Applications included the objective of enabling doctors “to access life-saving insights faster, through AI-assisted diagnostics” and advancing “the effectiveness of health interventions” and molecular structure predictions for drug discovery. The FDA provided non-legally-binding draft guidance in January 2025 related to Lifecycle Management and Marketing Submission Recommendations for AI-enabled devices (28). Lifecycle management was also promoted through the Digital Health Innovation Action Plan in 2017 (13), designed to diagnose misalignment with performance expectations in the field (12), although software updates altering system behavior can be costly to recertify (13).

Counterintuitively, the IVDR permitted AI use outside of intended scope if no better alternative existed, though with caution (27). Similarly, FDA market authorization was not required for internally developed AI solutions (20), although with severe risks if misused. Laboratory-developed tests (LDTs) were certified by the Clinical Laboratory Improvement Amendments (CLIA) in the USA (58), over which the FDA aimed to enhance authority, a move struck down, with LDTs deemed medical test services rather than devices (59). The UK's MHRA offered a Health Institution Exemption under Medical Devices Regulations 2002 when IVDs were developed and used exclusively in-house with “leading practice guidelines” (60), notably under the condition that no alternative existed (61).

### 3.1 Performance risks and monitoring

The EU and FDA introduced risk-based classification frameworks (28), with explainability encouraged for high-risk AI. In the EU, the Medical Device Regulation (MDR) software classification framework categorized non-diagnostic tools as Class I risk, tools such as physiology monitoring as Class IIa risk, systems associated with clinical deterioration risk as Class IIb risk, and life-threatening decision-making tools as Class III risk (13). The FDA reportedly designated WSI systems as highest risk at Class III (21), although we identified solutions at Class II (30, 62). Regulatory documents encourage human-in-the-loop (44) validation of high-risk decision-making during development and live scenarios (12). Decommissioning measures should be in place with “contingency plans to maintain essential services.” AI failure mode assessments have been provisioned by Microsoft and the open-source MITRE Adversarial Threat Landscape for AI Systems (ATLAS).

Recognizing a device's lifecycle benefits, risks (44), use case (12), and limitations is advised, along with post-market performance monitoring (28, 44). The FDA requires feedback on deaths, serious injuries, and malfunctions (28). Risks include overfitting, performance issues, input/output variability (44), data drift (28), and user errors (28). Data drift may be deployment-specific or related to data acquisition changes (28). Variability in pre-analytical processing, staining methods, and scanning can hinder AI applications (63), with calibration at deployment implemented as a possible mitigation (28, 31).

### 3.2 Cybersecurity and data security

Minimal cybersecurity testing is recommended in the form of malformed input (fuzz) testing and penetration testing (28). Threats include adversarial attacks, phishing, data poisoning, perturbation attacks, prompt injections, and AI hallucinations (12). Poisoned models may exhibit malicious behavior upon specific prompts. Perturbation attacks refer to stealthy AI input modifications, including prompt injection. Data poisoning mitigations include authentication and cleansing, anomaly detection, data integrity checks, adversarial training, and use of watermarks (28). LLM code has been deemed “inherently insecure” while risking arbitrary code execution (12). Nevertheless, secondary models may mediate as content and prompt filters, threat detectors, incident reporters, or security testers through mimicking. Open Worldwide Application Security Project LLM security risks, ATLAS, and “The Orange Book” are promoted as resources to assess failure modes.

Antithetically to the promotion of open-source models (12), risks are associated with attacker access to, or tampering with, model parameters and code. Library dependencies have been attacked; for instance, the Pickle format for model serialization has serious security flaws, risking arbitrary “opcode” execution during deserialization (64). Mitigations included use of trustworthy sources, avoiding pickle, loading model weights from TensorFlow and Flax checkpoints, or using a different serialization format (e.g., safetensors) (64). Pickle scanners are being implemented without guarantees. Regulatory sandboxes provide environments for testing technologies with fewer regulatory constraints (11).

Privacy advice included “data minimization” and adherence to the European Convention on Human Rights, UK General Data Protection, and the Data Protection Act 2018 (12), with risks reducible by anonymization and data security (13). De-identification methods include redaction, pseudonymization, and encryption (12); however, auxiliary data may still carry identification risks (42), potentially mitigated by the addition of “calibrated noise.” AI hosting platforms such as Amazon Bedrock, IBM watsonx.ai, and Microsoft's Azure OpenAI service offer zero-day retention policies (12). Federated learning involves local model training before a central update to reduce personal data sharing (42).

## 4 Histopathology WSI AI modeling logistics

**Figure 2:**
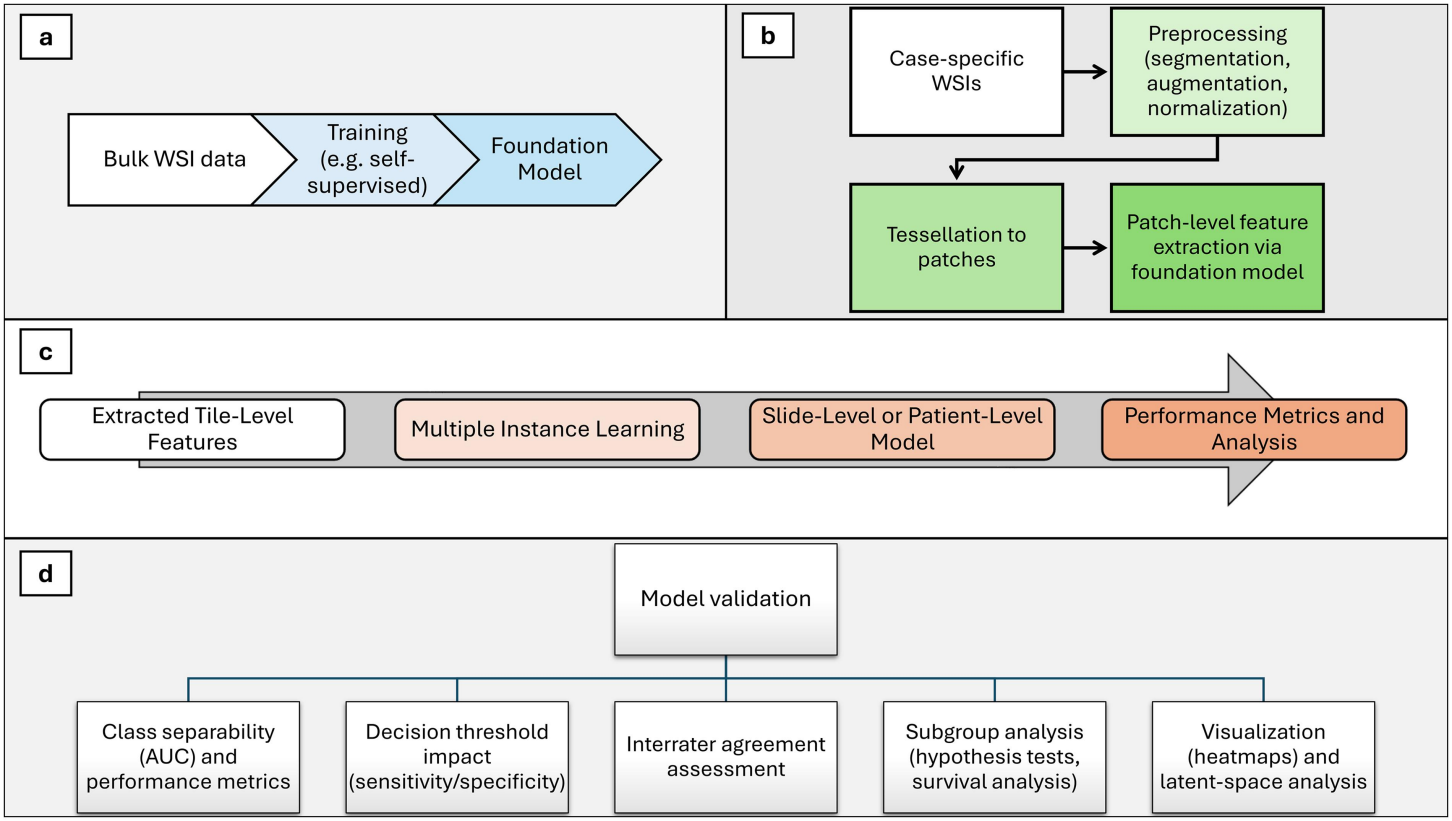
A logistical overview focused on the weakly supervised Multiple Instance Learning pathway. Modern research-only systems (104) typically process via two steps: WSI patch feature encoding, followed by slide encoding on those features. **(a)** A foundation model is trained on bulk WSI data, often using self-supervised learning. **(b)** The foundation model is used for tile-level feature extraction on case-specific WSIs following preprocessing, e.g., background removal segmentation, image augmentation, and normalization, followed by tessellation to tiles. **(c)** The high-quality extracted features are now available for slide- or patient-level modeling via Multiple Instance Learning (see Section 4.2.2). **(d)** The resulting classifier may be assessed by class separability and performance measures, sensitivity versus specificity, interobserver agreement with the classifier, analysis of stratified subgroups, visualization by heatmaps, and latent space analysis.

This section details pretrained foundation models (FMs), learning modalities, data acquisition, preprocessing, slide annotations, and validation methods (Figure 2). Supplementary Section S2 details hardware requirements.

### 4.1 Pretrained patch encoders for subsequent slide-level modeling

WSIs are often too large for direct AI modeling; however, dimensionality reduction of inputs by pretrained feature extractors can address graphical memory constraints (65). Training a feature extractor can take weeks and is deemed infeasible for every dataset. Feature extraction can be conducted on image patches (instances) (66) following WSI tessellation (22), with the impact of patch size remaining an open research question (18). Pretrained patch encoders are FMs for feature extraction at the patch or slide level, with slide-level FMs at risk of being undertrained (10). Pretrained FMs may mitigate the need for large datasets (22), with utility in low-data scenarios, since FMs generate transferable latent representations (embeddings) of the data, usable as inputs for decision-making modules such as transformer-based classifiers. Domain-specific feature extractors can enhance latent representations (18).

Textual data can augment vision encoder training, not only as fused inputs or generative outputs but also as semantic supervision. CONCH, based on contrastive captioners [CoCa (67)], is the state-of-the-art visual-language model for pathology (66) and is continually used in cutting-edge research (10). Contrastively trained on “histopathology images, biomedical text, and over 1.17 million image-caption pairs” (66), CONCH possesses image and text encoders, a multimodal decoder, and harnessed cosine similarity between encoder embeddings. Multimodal models developed using contrastive learning can be used in cross-modal retrieval (10), and contrastive learning provides self-supervised learning (SSL) enhancements to the encoders.

A total of 19 feature extractor FMs were benchmarked upon H&E slides across lung, colorectal, gastric, and breast cancer for weakly supervised tasks (22) across morphology, biomarkers, prognostication, mutations, and phenotypes. Of these, 12 models were vision-only, 3 vision-language, and 4 slide encoders. CONCH was the highest performer, with Virchow2 a close second; an ensemble of both achieved state-of-the-art performance. For downstream tasks following feature extraction, both transformer-based aggregation and attention-based multiple instance learning (ABMIL) were considered. ABMIL used attention to assign weights to tiles. Transformer-based Solid Tumor Associative Modeling in Pathology (STAMP) architecture was found to marginally outperform ABMIL.

### 4.2 Learning modalities

This section discusses key AI histopathology modeling modalities, including SSL, transfer learning, few-shot learning, fine-tuning, multiple instance learning (MIL), ensembles, mixture of experts (MOE), and concatenation usage (Supplementary Section S3). Incremental learning (also referred to as continual or lifelong learning) was proposed in the Queryable Prototype MIL framework for WSI classification (66) (Supplementary Section S3). This involves sequential training over multiple datasets to mitigate “catastrophic forgetting,” motivated by the dynamic nature of data over time.

#### 4.2.1 Self-supervised learning, transfer learning for few-shot learning, and fine-tuning

SSL can be used to develop models with high-quality latent representations by training on a “pretext task” (31). SSL can be conducted via autoencoders, variational encoders, masked image reconstruction, or contrastive learning (10, 18, 22), resulting in greater performance with fewer parameters than competitors (10), without requiring labels. Fewer network layers can be more performant, and vision-language contrastive pretraining enhances performance (10), substantiated by CONCH performing at least on par with the vision-only Virchow2 (22). Unimodal contrastive learning between augmentations of the same image is also possible (31). Models with rich latent representations developed using SSL are deemed suitable for identifying candidate biomarkers when applied with clinical or molecular labels (20).

Transfer learning is recommended for generalizing to scarce data problems (47). In the development of research-only Transformer-based pathology Image and Text Alignment Network (TITAN), visual SSL was followed by CoCa-based pretraining with caption alignment on fine-grained morphological descriptions, and subsequently with coarse clinical reports of microscopic findings (10). TITAN demonstrated excellent few-shot learning, with implications for use in rare, low-sample cancer settings. “Linear probe evaluation” used a linear layer for transfer learning, with few-shot learning assessed via the “SimpleShot” framework.

LoRA (low-rank adaptation), or QLoRA, is available to fine-tune FMs potentially containing trillions of parameters (68), drastically reducing trainable parameter requirements for model repurposing. FM weights and parameters are frozen, while trainable “lower-rank matrices” are introduced to modify network behavior at its various layers by gradient descent. The “parameter-efficient fine-tuning” library is available to PyTorch developers. Due to its linearity, following training, LoRA weights can be merged with the pretrained model. LoRA can produce information loss, often insignificant because DL models are overparameterized. LoRA can also be used alongside prefix-tuning. LLM augmentation using retrieval-augmented generation avoids full training or fine-tuning, making it useful for interpreting documents under dynamic change (12).

#### 4.2.2 Multiple instance learning

MIL is an ML approach applicable to images too large to process through conventional methods. It treats a WSI as a “bag” of image patches, individually encoded by a feature extractor, with patch encodings aggregated to form a prediction (18). It uses slide-level annotations, which are more routinely collected than region-, patch-, or pixel-level annotations. This constitutes a “weakly supervised” ML problem. Patches are commonly used in a non-overlapping fashion (25, 69–71). By contrast, segmentation models require local annotations and considerable manual work (18). Despite weak labeling, MIL is amenable to localized interpretability.

Patch encodings resemble tokens in sequence generation models, where multiple latent vector representations operate as fundamental units. Following patch encoding, patch-level embedding vectors are stacked to form a feature matrix (18). This matrix can, in principle, then be aggregated or pooled by any function capable of projection. Consequently, contextually aware transformer models are appropriate for aggregation (10, 25), although potentially expensive for feature extraction (18). Graph neural networks have also been proposed to represent the embedding matrix, with connectivity dependent on embedding distances in high-dimensional space.

MIL can operate on scalar or vector patch representations, termed instance-based and embedding-based, respectively (18). The instance-based approach forms a scalar score or confidence at the patch level, as derived from the projection of a feature extractor into a single output node. While instance-based MIL provides immediate patch-level interpretability, embedding-based MIL captures powerful representations for decision modules. Studies have also attempted to combine the methods. Interpretability of embedding-based methods has used multiple region-of-interest (ROI) subsampling to generate confidence maps.

#### 4.2.3 Ensemble approaches and mixture of experts

AI ensembles work analogously to human pathologists reaching consensus on a common task (46), for example, the “Delphi method” (6). Ensemble approaches may reduce biases and overfitting while enhancing generalizability by combined knowledge (21). A three-model CNN ensemble associated with Ibex's Galen Second Read (GSR) was used in clinical practice (41), and an ensemble was used by the leader of CGC17 for grading lymph node (LN) involvement in cancer (72). Ensembles were also used in the CE-IVD-marked DoMore! Project (34, 69–71). Human–AI perspectives can also be combined; for example, MSIntuit AI predictions were combined with MSPath to measure tumor MSI probability, with MSIntuit outperforming the latter (31). Combining predictions using a “dichotomic classifier” increased sensitivity, albeit decreasing specificity considerably. In research, pretrained patch encoders were enhanced through usage as an ensemble (22). Two approaches were taken: (1) concatenation of feature vectors for a unified prediction model and (2) prediction score averaging. The latter was more successful with averaging the top four models, although concatenation was also robust.

The ensemble concept differs from the MoE framework, which uses “conditional computation” to route data to specialist models via a gating network (router), thereby significantly reducing computational costs, albeit not memory requirements, while even enhancing performance (73). Within LLMs using MoE, routing can occur at multiple layers. Fine-tuning MoEs presents greater difficulty than standard networks.

### 4.3 Datasets

The literature discusses many datasets related to histopathology AI (10). FDA guidelines highlight the importance of quality, quantity, and diverse data for AI development (28). Data diversity and representation can be enhanced (21) by using heterogeneous data (20) across disease stages, tissue types, demographics, and image collection methods (21). Consequently, collaboration across multiple sites and organizations is advised. Greater amounts of pretraining data are associated with better model performance (10, 22), while data diversity has been shown to be more importance than volume (22). A comparison of data scales is highlighted in Table 3.

**Table 3:** Examples from across the review for research-only and marketed solutions to illustrate data scale in training, testing, external validation, and calibration.

Application	Data	Usage	Market-approved WSI AI platform?
CAMELYON16	400 H&E WSIs (40)	Metastasis detection competition	No
CONCH	1.17 million image–caption pairs (66)	Contrastively trained feature extractor	No
Histotype Px Colorectal	4,500 patient samples and 90 million image tiles (34)	Training and testing	Yes
Ibex Prostate Detect	∼1.36M image patches from 549 manually annotated slides, internal test dataset of 2,501 slides, external dataset of 1,627 slides (41)	Training and validation for Prostate cancer detection and Gleason grading	Yes
Lunit SCOPE PD-L1	>1,000,000 cancer cell images (102), 393,565 annotated tumor cells, 4,675 IHC stained tissue grids from 802 WSIs (36)	PD-L1 biomarker TPS analyzer training	Yes
MSIntuit	4 million TCGA Colon Adenocarcinoma tiles (31)	50-layer ResNet50 feature extractor trained by Momentum Contrast used in MSI Binary Classification	Yes
MSIntuit and Galen Prostate	30 MSI slides/cases (31, 41)	Device calibration	Yes
Paige Prostate	232 H&E WSIs and 579 WSIs (46)	External validation	Yes
PRISM	16 tissue types, 587k H&E WSIs, 195k clinical reports (96)	Multimodal vision-LLM built upon Virchow	No
TITAN	335,645 WSIs across 20 organs and 182,862 medical reports (10)	iBOT Vision training + contrastive vision-language pretraining	No
Virchow	∼1 million slides on 17 tissue types (96)	Vision-only feature extractor	No
Visiopharm Metastasis Detection app and DeepPath LYDIA	WSIs from 455 patients, 6 tumor types (27)	Retrospective within-distribution and out-of-distribution assessment	Yes

Acquiring sufficient, good quality data is fundamental to AI engineering. This table highlights the diversity of sample reporting and usage dependent on implementation, where in many cases the datasets are unreleased. This includes counts across WSIs, tiles, cases, tissues, medical reports, cell annotations, specific stains, and image–caption pairs. Usage spanned model training for detection, classification, and feature extraction; vision-language and multimodal model training; internal and external validation; biomarker quantification; device calibration; and within- and out-of-distribution assessment.

The Cancer Genome Atlas (TCGA) (74) is a major medical data repository, including WSIs (Supplementary Section S5 details an acquisition method). However, massive propriety datasets have seen increased use (22). The Clinical Proteomic Tumor Analysis Consortium project data were used in WSI-related investigations (22), aimed at understanding cancer through proteomics (75). Related to Artera Prostate, the NRG Oncology Biobank provided large and diverse prospective trials across hundreds of centers for multimodal AI development (62). For TITAN (10), a dataset of 25,495 tumor-containing regions at 8 K resolution, ×20 magnification, with 32 classes (76), was fused with the EBRAINS brain tumor dataset (77) and released as the pan-cancer “Rare-Cancer-Public.” An associated TCGA-derived, pathologist-curated dataset (>1.6 million H&E 256 × 256 image patches) covering 32 different cancer types was also made available (78, 79).

The CAMELYON16 dataset (600 GB, 400 H&E WSIs from two medical centers) (40), annotated at pixel level, was released to stimulate research into automatic breast cancer metastasis detection in sentinel LNs (65). The objective of CGC17 was to determine breast cancer lymph node staging (pN) related to isolated tumor cells, micro-metastases and macro-metastases (40), while shifting to clinically applicable multi-slide patient-level analysis. The website provided a leaderboard and short papers. The leading CGC17 project was developed by Deep Bio Inc. (72), a company associated with CE-marked DeepDx Prostate (2). The solution used CAMELYON16 and CGC17 data along with “auto hard mining” and a modified Deeplab v3+ patch classifier to produce pixel-level classifications and heatmaps (72). Auto hard mining enforced training on the hardest examples, achieved by focusing on patches with low intersection-over-union scores relative to ground-truth pixel-level annotated segmentation masks. Three checkpoints from different folds were ensembled. Augmentation included randomized color jitter, flip, and rotation. DBSCAN clustering grouped tumor regions on heatmaps, with metastasis classification determined by measuring the biggest tumor region's longest axis.

### 4.4 Preprocessing

Regulatory guidelines highlight the need to document preprocessing steps (28). This section considers slide artifacts and normalization, data augmentation, imputation (Supplementary Section S6), segmentation, and slide annotation.

#### 4.4.1 Artifacts and stain normalization

Besides scanner variability, pre-analytical variables such as poor sample quality, staining artifacts, and air bubbles can adversely affect pathologist and AI performances (63). In the external validation of Artera Prostate, an artifact classifier filtered out low-quality images (80). The CE-IVD-marked MSIntuit included slide checking as part of its clinical deployment strategy to identify slides containing “large blurry regions” and other artifacts such as slide pen marks and background noise (31). These blurry regions were undetectable to pathologists, and AI performance decrease was noted when excluding this step. Stain normalization can be conducted (18), though this practice could be obsolete in view of improving FMs for pathology (25), with generalization and normalization across data sources and scanners actively researched (24). Furthermore, overly clean data may not reflect real-world diversity (36). Some studies ignore stain normalization completely (22).

#### 4.4.2 Data augmentation

A strategy to enhance generalizability is data input augmentation through small transformations (47), with histopathology likely to benefit from rotational invariance. Offline data augmentation may be infeasible due to the sizes of datasets (10). Achievable via “distortion algorithm” (71), augmentation was conducted on-the-fly for PD-L1 cell classification, with rotation, scaling, vertical/horizontal flipping, shearing, elastic deformations, blurring, random cropping, and alterations to color and intensity characteristics (36).

#### 4.4.3 Segmentation and annotation

Segmentation is used to identify relevant tissue regions on a slide (18). The CLAM toolbox can conduct tissue segmentation and tiling prior to feature extraction (10, 66). OpenSlide and openslide-python also provide WSI preprocessing (10). Slide background tiles can be removed using the Canny edge detector (22), while Otsu thresholding facilitates segmentation of the tissue from background as well as contouring (24). For Galen Prostate, a Gradient Boosting classifier identified tissue from background (41).

Semantic segmentation provides labels to pixels, while instance segmentation labels objects separately. Semantic segmentation is achieved by annotating WSI regions with classes. Segmentation can be used within ROIs for quantification, aiding prognosis and treatment response assessment (9). Accurate histological segmentation has been deemed important for image-based biomarker discovery in WSIs (81), though often considered “laborious” and potentially “intractable.” For MSIntuit, a U-Net CNN was trained for segmentation on 460 H&E and IHC manually annotated slides and validated on 115 slides (31). The Dice score (0.96) indicated high overlap between the predicted segmentation mask and the true mask, with the ground-truth mask typically derived from semi-automated or manual expert segmentation (9). Slide checking was followed by feature extraction, and these features were run through an MLP to estimate tumor proportion and discard slides with few tumor-positive tiles (31). Beyond WSIs, U-Net was the most utilized architecture for lymphoma lesion segmentation in Positron Emission Tomography (PET) and Computed Tomography (CT) (9), with commercial lesion detectors available via Siemens Healthineers.

**Figure 3:**
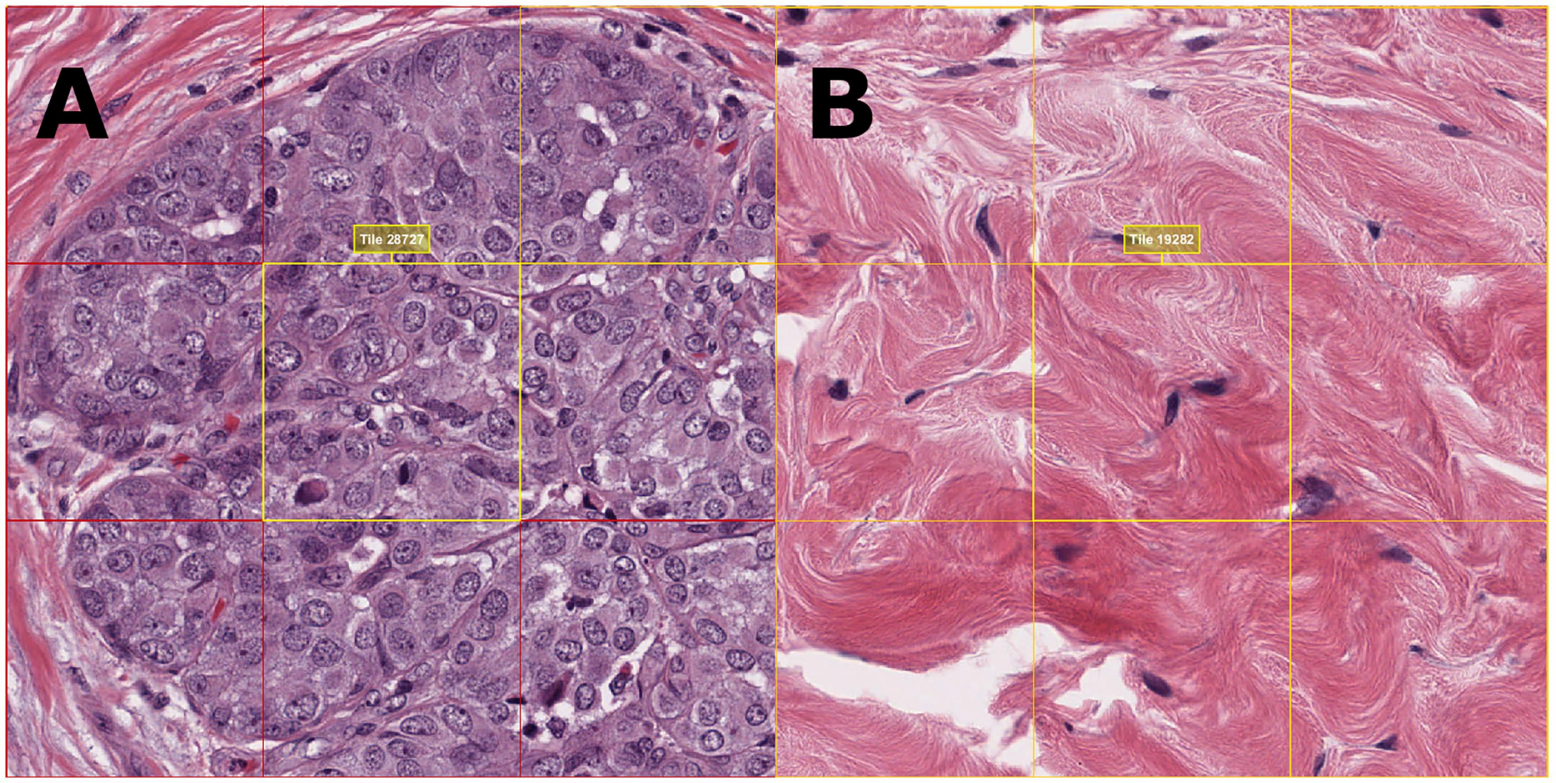
Qupath WSInfer AI tool usage illustrated as two 9-tile crops on a TCGA-BRCA slide using the breast-tumor-resnet34.tcga-brca model. **(A)** Center tile strongly positive for tumor with dense cell clusters, large nuclei (hallmarks of rapid mitosis), and loss of structure. **(B)** Strongly negative for tumor according to the model, with visible structural integrity and normal nuclei.

Effective and extensive expert annotation has been critical for pathology AI model development (24). The hierarchy of labeling may be at the pixel, patch, specimen, cell, slide, or patient levels (24, 26, 38). Weak supervision is expected to require more data than strong supervision (24, 25), while also providing limited ROI information for ML (21). Strong, fully supervised learning requires labor-intensive and error-prone pixel-wise annotation (21). Pixel- or patch-level annotation can be achieved by drawing contour regions on WSIs (24). This appears to have been used for Galen Prostate, with slide region annotations subsequently fragmented into patch samples (41). Strongly supervised approaches lend themselves to tumor localization within a slide for classification (65), whereas weak supervision is suitable for survival, molecular subtype prediction, or transcriptomic gene expression where no localized labeling intrinsically defines the outcome. This perspective may understate the potential of morphological region annotation for quantification strategies. Extensible and open-source QuPath (24, 82) possesses an extension manager with readily downloadable AI tools that we tested on TCGA breast cancer slides (Figure 3). InstanSeg segmented nuclei, and WSInfer probabilistically classified tiles.

Iterative human–machine workflows were developed (e.g., Aiforia or QuickAnnotator) to incrementally improve an annotation guidance model in real time, although with bias risk (24). Quick Annotator combined live updates of a U-Net DL model with ongoing user annotations (81). Users could accept or correct suggestions to produce binary masks, with diverse features selectable from a UMAP clustering plot for user curation. Annotation of nuclei, tubules, and epithelium was performed 102×, 9×, and 39× faster, respectively. The baseline model was developed using self-supervised autoencoding followed by supervised fine-tuning for segmentation. The results reported strong f-scores in Quick Annotator-produced masks, with the capacity to annotate with precision intractable for a human.

Owkin are MHRA-registered (83) for image analysis interpretative IVD software, with CE-IVD-marking for the diagnostic MSIntuit AI tool (84). An Owkin-associated study discussed how SSL using Momentum Contrast v2 coupled with weakly supervised learning closed the performance gap with strong supervision (65). By employing augmentation through dual encoders, this contrastive approach pushed similar tiles together and different tiles apart in the learned embedding space. At the time, strongly supervised approaches were considered state of the art, while weakly supervised approaches tended to use out-of-domain ImageNet-based feature extractors. Consequently, they developed an in-domain feature extractor, now commonplace within state-of-the-art pipelines (10, 25), coupled with multiple downstream MIL approaches (Chowder, Weldon, and DeepMIL) (65). State-of-the-art AUC was achieved on Camelyon16, increasing from 91.4% to 98.7% under weak supervision, although not reaching the 99.3% supervised performance benchmark. Its feature extractor provided much higher AUC for feature clustering (*k* = 10) than ImageNet. Cluster mappings were overlaid as heatmaps on Camelyon16 slides to demonstrate superior grouping for tumoral tiles, with one cluster especially correlating to tumors. Feature extractors for TCGA-COAD transferred well to Camelyon16, but not vice-versa, possibly due to TCGA-COAD data originating from more sites and greater natural variation in colorectal cancer.

### 4.5 Validation metrics and methods

The importance of metrics in AI systems development was noted in UK government guidelines (12). With yearly increases in approvals, the FDA-approved 1,016 AI-based medical devices as of October 2024, the majority radiology-focused (13). Many of these devices were reported to have limited validation of longitudinal robustness, with an emphasis on accuracy. Thus, this section details metrics and validation methods related to histopathology AI. Language model evaluations and confidence intervals are detailed in Supplementary Sections S7, S8.

#### 4.5.1 AUC limitations and concordance

Multiple patch-level feature extractors have been compared using AUROC, area under the precision–recall characteristic (AUPRC), DeLong's test, balanced accuracy, and F1 (22). Balanced accuracy is the average of sensitivity and specificity (48), and is used in multiclass WSI classification, with AUROC used in binary tasks (10). While most studies emphasize classification, continuous outputs can be assessed using the coefficient of determination (R^²^) (85), mean absolute error, root-mean-squared error, scatter plot, Deming regression, Bland–Altman analysis, decision curve analysis, calibration plots, and predictiveness curve analysis for biomarker evaluation (28).

The area under the receiver operating characteristic (AUROC or AUC) (28) measures the ability of a model to distinguish between predicted labels (25, 48). The AUPRC is considered better for handling imbalanced data (25). AUC and AUPRC score maximally at a value of 1. A 2022 assessment of DL papers on MSI or DNA mismatch repair deficiency (dMMR) prediction identified that most studies emphasized AUC while neglecting sensitivity and specificity (48). AUROC was considered “not relevant to clinical practice” (31) as it “can hide a severe lack of generalization” (31, 48), as discussed in relation to MSIntuit validation (31). Sensitivity, specificity, and Negative Predictive Value (NPV) were preferred.

AUC for binary tasks is calculated by modulating the decision threshold and extracting sensitivity and specificity (48). Thus, AUC measures class separability by ranking output values irrespective of the final chosen decision boundary. Consequently, AUC is unaffected by linear scaling of the outputs, risking external data misclassification under calibration loss. The same limitation applies to concordance indices (c-index), which represent a generalization of AUC for time-to-event data. A mitigation strategy is threshold calibration at deployment, although uncertainty on the threshold means that generalizability is preferred. A study validating GSR termed decision thresholds as “somewhat arbitrary” (32). The MSIntuit team included a sensitivity-focused calibration step to enhance clinical applicability (31); however, the 30 MSI slides required for calibration, as determined by sensitivity analysis, were seen as a limitation for small centers. Galen Prostate also calibrated on 30 cases for new laboratories (41), although with aspirations for enhanced generalization across scanners and laboratories. Decision threshold calibration should not be confused with Expected Calibration Error (10), which measures the weighted average of the absolute difference between the true proportion of positives and mean prediction probabilities across a predefined number (*K*) of prediction probability bins (86).

In survival analysis, the c-index measures whether the order of patient events corresponds to the order of predicted risk (87). Harrell's c-index has been used to optimize risk stratification systems (35), and to evaluate transfer learning using linear probes for survival tasks (25). However, “concordance analysis” also refers to interrater agreement, which may be reported by simple regression between raters or Bland–Altman plots that represent interrater reading differences versus mean readings within agreement limits (88).

#### 4.5.2 Interrater agreement and hypothesis testing for subgroup analysis

Cohen's *κ* is used to measure concordance between two raters; for instance high-performing FMs demonstrated higher agreement than low performers (22). It is used on categorical data and measures observed agreement (perfect agreement Cohen's *κ* = 1) versus chance (Cohen's *κ* = 0) (88). Consequently, Cohen's *κ* can be used to assess AI agreement with a consensus reference (2). In the validation of Genius cervical AI, Kendall W coefficient was used to assess consistency between three pathologist users, and routine screening was contrasted with AI-guided interpretations using the *κ* coefficient (51). Fleiss' *κ* (31) is used to assess agreement between two or more evaluators or methods (89). The intraclass correlation coefficient can also be used to assess interobserver agreement (37). Bland–Altman plots can display interobserver concordance, with limits of agreement also reported (28).

Hypothesis testing was proposed by the FDA to compare against a “pre-specified performance goal” (28). Hypothesis tests may be used to evaluate for differences between subgroups within a validation cohort (80), for instance, Wilcoxon rank-sum test, Fisher's exact test, and Pearson's chi-square test. Bonferroni correction was recommended for studies with multiple comparisons (47). McNemar's test (31) was used to compare methods using paired nominal binary data (90). The Spearman rho test can be used to compare clinician–device, device–device, or clinician–clinician performance in tumor proportion score (TPS)-based classification (36). Pearson correlation coefficients are reported for interobserver agreement, including between human and AI (41).

#### 4.5.3 Survival analysis

Survival analysis can evaluate time-to-event separability between groups determined by AI classification (80). Cox proportional hazards (CPH) models are used to assess survival clinical endpoints, implementable with the scikit-surv package (10). The performance of Stratipath Breast prognostic prediction AI was evaluated using multivariate CPH analysis to assess relative risk over time for progression-free survival (23). The proportional hazards assumption was assessed using log-log plots (71). Fine-Gray sub-distribution hazard modeling is used to estimate cumulative incidence of events under competing risks (91), and was used to assess endpoints to yield sub-distributional hazard ratios (sHRs) (80).

Kaplan–Meier curves represent % survival probability over time (23, 35) and have been used to estimate cancer-specific survival at specific time points (35). They illustrate survival probabilities over a time -course for stratified groupings (25). However, Kaplan–Meier may bias cumulative incidence estimates if competing risks are censored (91). The Mantle–Cox log-rank test is a statistical test for comparing survival distributions and was used to assess whether AI patient risk classification reflected cancer-specific survival (35, 71).

#### 4.5.4 Sensitivity vs. specificity

Sensitivity, (TP/(TP+FN)), is a relationship between true positives (TP) and false negatives (FN) (1). Specificity, (TN/(TN+FP)), is a relationship between true negatives (TN) and false positives (FP). Confusion matrices visualize FPs and FNs (12, 28), providing insights into edge cases for subsequent fine-tuning (12). Sensitivity, specificity, positive predictive values (PPV), and NPV are related concepts (28). Positive and negative diagnostic likelihood ratios (PLR and NLR, also denoted LR+ and LR−) can also be considered. Likelihood ratios are noted for risk stratification models that perform risk and prognostic group classifications.

Asymmetric verification occurs when only negative H&E diagnostic tests undergo confirmatory IHC testing for IHC cost reductions (4). Consequently, specificity and PPV cannot be estimated as FPs are unidentifiable. Supported by prior reports of low FP rates, high sensitivity and NPV are prioritized to avoid FNs, under the assumption that screened positives represent ground truth. However, many CE-marked commercial devices substantially generate FPs even with usage within distribution (27). Nevertheless, emphasis on case-level sensitivity is deemed the “basic premise for AI use.” In a Paige Prostate study emphasizing high sensitivity and NPV, the solution was determined to “safely decrease pathology workload without compromising diagnostic quality” (38). A similar approach was taken with CE-IVD-marked MSIntuit to reduce MSI testing burden in colorectal cancer (31). Such high sensitivity screening approaches support perspectives positioning future pathologists as AI validators, with full automation on non-malignant cases (21).

While it is important that AI diagnostic methods reduce conventional non-AI FN rates, for instance 1%–4% for prostate needle biopsies (38), elsewhere estimated between 1% and 10% in prostate cancer (32), the burden of FPs needs careful evaluation at the patient level, given the risk of unnecessary treatment. Furthermore, FP alerts may represent an annotation burden and are understated by commercial documents by up to fivefold (27). Segmentation FP alerts may falsely flag blood vessels, histiocytes, follicle centers, capsular naevi, and nerves (4, 27). GSR set a threshold to balance sensitivity and specificity (32), acknowledging the inevitable burden of FPs associated with their sensitivity emphasis. A total of 1,733 out of 4,385 cases that were diagnosed as benign by pathologists were given AI cancer alerts, but only 128 required revision. Nevertheless, the AI-guided second read was likely a faster process and beneficial in reducing net FNs. Gleason grading also underwent similar correction.

#### 4.5.5 Visualization

Visualization provides vital interpretability (36), with heatmaps being widely used. Heatmaps can visually illustrate how attention heads focus on different histological structures, ranging from dense tumor to non-tumor regions (10). Attention heatmaps have been generated using Grad-CAM (22). FDA-certified Paige Prostate generated heatmaps of high-probability regions suspicious for cancer (38). CE-IVD-certified Visiopharm highlighted metastatic probabilities with outlines derived from probability distributions produced by SoftMax (4), resembling a traffic light system (29). The red outline represented an optimal balance of sensitivity and specificity. Individual cells assessed for biomarker presence can be highlighted, including via bounding box (36).

The CE-IVD-certified MSIntuit tool used tile-level scores to highlight most predictive regions on heatmaps (31). Furthermore, a ResNet18 segmentation model from the TIAToolbox library was used to map eight tissue categories to the slides: adipose, debris, lymphocytes, mucin, smooth muscle, normal colon mucosa, cancer-associated stroma, and colorectal adenocarcinoma. Normal glands were non-MSI predictive, while MSI-predictive tiles contained tumor cells, inflammation, mucin, poor differentiation, and tumor-infiltrating lymphocytes (TIL). However, mucin was also associated with FPs. MSI predictive regions were often located outside the tumor area. Another AI had FPs triggered by glands (32). Indeed, “cancer mimics” are recognized AI confounders (41).

Ibex Prostate Detect (GSR) used heatmaps with quality control alerts (32, 39), and their breast cancer solution generated contours and cell overlays (37). Galen Prostate produced probability heatmaps for cancer presence, Gleason grading, perineural invasion, high-grade intraepithelial neoplasia, inflammation, atrophy, and benign tissue (1). Galen Breast displayed cancer presence, invasive ductal carcinoma versus invasive lobular carcinoma, atypical ductal hyperplasia (ADH) vs. ductal carcinoma *in situ* (DCIS), DCIS grading, lymphovascular invasion, TIL, and other findings.

#### 4.5.6 Latent space analysis

Dimensionality reduction techniques, such as UMAP, have been used to visualize AI-encoded latent representations across organs, populations, and data acquisition methods to highlight feature diversity and generalizability (10, 66, 81). Such approaches can reveal separability associated with intraclass variations, disease stages, recovery or immune profiles, prognoses, and therapeutic responses. Unsupervised approaches have also been proposed for identifying novel biomarkers and disease subtypes (21), although challenges remain regarding translational interpretability and validation.

## 5 Companies with regulatory-approved solutions

This section discusses literature associated with regulatory-approved solutions. Although many of these studies involve competing interests (4, 23, 27, 35–38, 41, 80), they also represent breakthrough translational engineering. Table 4 summarizes companies and their flagship products discussed in this section.

**Table 4:** Countries of origin, flagship products, identified approval (FDA or CE) status, uses of flagship products, and associated study metrics for companies that developed market-approved WSI AI as identified in this review.

Company	Country of origin (approval identified)	Flagship product	Use of flagship product	Associated study metrics
Artera	United States (FDA)	ArteraAI Prostate	Prostate cancer prognostic risk decision support	External validation on 318 men; fine-Gray sHRs 2.33 and 3.54 for primary endpoints
Deep Bio Inc.	South Korea (CE)	DeepDx Prostate	Prostate cancer grading	Cohen's *κ* 0.91 for cancer vs. non-cancer; sensitivity 0.997, specificity 0.88, NPV 0.99, PPV 0.95. Gleason grading: weighted *κ* 0.89, unweighted *κ* 0.56
DeepPATH	United Kingdom (CE)	DeepPath LYDIA	Colorectal, breast, lung, and melanoma lymph node assessment	In VMD/DPL comparison: 100% sensitivity for colorectal and breast macro-metastases; 95.8% for micro-metastases; 44.4% for breast ITCs; DPL showed 100% out-of-distribution sensitivity in head and neck cancer
DoMore Diagnostics	Norway (CE)	Histotype Px Colorectal	Colorectal prognostic risk assessment	HR 10.71 between high- and low-risk groups in one analysis; adjusted HR 3.04 between poor and good prognosis; 3-year survival classification sensitivity 52%, specificity 78%, PPV 19%, NPV 94%, accuracy 76%
Hologic	United States (FDA & CE)	Genius™ Digital Diagnostics System with the Genius™ Cervical AI algorithm	Cervical screening	Independent validation on 890 ThinPrep Pap tests; sensitivity 98.2%–100% for atypical squamous cells of undetermined significance or higher classification; NPV 92.4%–100%; Kendall W 0.909; review time 37–77 s vs. 3–5 min
Ibex Medical Analytics	Israel (FDA & CE)	Ibex Prostate Detect	Prostate cancer detection and Gleason grading	Slide-level detection: specificity 96.7%, sensitivity 96.6%, AUC 0.994, NPV 99.2%. Grade 1 vs. higher: specificity 82.1%, sensitivity 81.1%, AUC 0.901. Foundational external cancer detection: AUC 0.991, sensitivity 98%, specificity 97%, NPV 99.1%
Lunit	South Korea (CE)	Lunit SCOPE PD-L1	PD-L1 biomarker tumor proportion score analyzer	Validation on 430 external WSIs; TPS concordance 85.7%, 89.3%, and 52.4% for <1%, 1%–49%, and ≥50%; AI–pathologist Spearman coefficient 0.925
Owkin	France (CE)	MSIntuit	Colorectal pre-screening for DNA mismatch repair deficiency and Microsatellite Instability	Blind validation on independent 600-patient dataset: sensitivity 0.96–0.98, specificity 0.46–0.47. Inter-scanner Cohen's *κ* 0.82; tile-level score correlation R = 0.92
Paige	United States (FDA)	Paige Prostate	Prostate cancer foci detection, Gleason grading, tumor burden quantification, perineural invasion assessment	Out-of-distribution validation: AUC 0.96, sensitivity 91%, specificity 94%. External validation: part-specimen sensitivity 99%, NPV 100%, specificity 93%; patient-level sensitivity 100%, NPV 100%, specificity 78%; 65.5% diagnostic time reduction
Stratipath	Sweden (CE)	Stratipath Breast	Breast cancer prognostic binary classification	Validated against two independent cohorts; multivariate CPH HR 2.76 for progression-free survival between high- and low-risk ER+/HER2− groups; HR 2.20 when including Nottingham grade 2
Visiopharm	Denmark (CE)	Visiopharm Metastasis Detection app	Colorectal and breast cancer lymph node assessment	Visiopharm Integrator metastasis algorithm: sensitivity and NPV 100%. Standalone VMD: sensitivity 95.8%; detected all macro-metastases; ITC sensitivity 44.4%

Note that final market approval validation reports, as can be found on FDA websites, can differ from study reports. As noted throughout the text, EU pathway evidence is less transparent.

Companies may seek to produce a unified AI platform. Roche (5) described the “Digital Pathology Open Environment,” which combines Roche's and a third-party AI’s image analysis algorithms. Notable CE-IVD-marked solutions include MSIntuit CRC resection H&E by OWKIN, Ibex (Ibex Prostate) (92) also associated with FDA approval (49), Stratipath Breast (23), and DeepDx Prostate by Deep Bio (2). Other solutions include Kwant Her 2 by DiaDeep, Lunit SCOPE PD-L1 22C3 TPS, GD-L1 Gastric by Mindpeak, PathAI PD-L1 (28-8) NSCLC, Qai Prostate Grade by QRITIVE, uPath Ki-67 30-9 image analysis by Roche, and Sonrai Research MSI (5).

### 5.1 Paige

Paige Prostate achieved Breakthrough Designation (38) and was granted approval by the FDA in September 2021 (93), a first for AI digital pathology. It was approved as a Class II device (30), applying FMs to cancer care (26). Paige Prostate also won the NHS Artificial Intelligence in Health and Care Award in 2021 (94). More contemporary to Paige Prostate Alpha, Paige Prostate 1.0 was developed as a CNN with MIL designed for ×20 resolution (38). Paige Prostate detected cancer foci in H&E WSIs prepared from needle biopsy (95), predicted Gleason grade, quantified tumor burden (26), and assessed prostate perineural invasion by identifying suspicious perineural foci. Consequently, slide evaluation and diagnostic times were down 21.9% and 65.5%, respectively, with a reported 70% reduction in diagnostic error.

NICE published an innovation briefing on Paige Prostate in November 2021 (46), highlighting the innovative use of MIL with high sensitivity and throughput, but concluding that evidence was low to moderate, with a further need for UK population studies. Five observational studies were noted, highlighting the possibility of trialing AI in parallel with ongoing clinical practice. One study utilizing Paige Prostate Alpha reported pathologist sensitivity increase from 74% to 90%, with no significant specificity change on 232 H&E WSIs; it particularly noted enhanced smaller, lower-grade tumor detection. In 2020, Paige Prostate 1.0's out-of-distribution generalization was evaluated on neoadjuvant-treated prostate tissue, resulting in AUC 0.96, sensitivity 91%, and specificity 94%. This low sensitivity was deemed a risk for FNs, requiring expert consensus.

An external validation study aimed to classify slides into benign or suspicious to flag for further histological or immunohistochemical analyses (38). The AI demonstrated strong generalization on an overseas external cohort. Ground truth was based on human–AI concordance, including IHC confirmation for high-molecular-weight cytokeratin, p63, and P504S on randomly selected slides. Paige Prostate demonstrated 99% sensitivity for part specimens (CI 96%–100%), 100% NPV (CI 98%–100%), and specificity 93% (CI 90%–96%). At the patient level, it demonstrated 100% sensitivity (CI 93%–100%) and 100% NPV (CI 91%–100%), with specificity falling to 78% (CI 64%–89%). Four diagnoses were corrected to malignant from benign using the AI solution, along with a 65.5% diagnostic time reduction across 579 WSIs.

Paige also provided Paige Breast and Paige Breast Lymph Node (PBLN) (63). PBLN utilized the prostate cancer detection technology for LN assessment. Paige Breast held FDA Breakthrough Designation for cancer identification and classification from H&E WSI LN specimens (26), with potential cancer foci, pre-cancerous neoplasm detection, and mitotic counting within mitotic hotspots. Paige also developed HER2Complete assay, which measured Human Epidermal Growth Factor Receptor 2 (HER2) expression levels from H&E. Paige GI Suite was advertised to support pathologists in detecting and classifying GI tract benign and malignant conditions applying FMs on biopsies and resections across multiple sites, such as the pancreas, stomach, and small intestine. It identified suspicious colon carcinoma foci, high-grade dysplasia, high MSI, and dMMR phenotypes.

Paige also offered customized AI, with FDA Breakthrough Device Designation granted to Paige PanCancer, designed to identify suspicious cancer regions across 21 different organ biopsies and 25 tissue resections. At the specimen level, AUC 0.95 was reported for common and rare cancers. The PanCancer Suite used Virchow, with FMs eliminating the need for “task-specific training.” Virchow was the first million-slide FM for cancer and operated on 17 tissue types (96). Virchow2 was trained across H&E and IHC, with reported precision enhancements across 40+ tissue types. Virchow2G was the largest model (1.8B parameters) and Virchow2G-Mini was its lightweight alternative. The 2G variants were available under commercial license only. Virchow, Virchow2, and PRISM were available via Hugging Face and Azure AI Foundry. Their website also listed PRISM, which was built upon Virchow and represented a multi-modal vision-LLM for WSI analysis, clinical report generation, and zero-shot cancer classification. PRISM was trained across 16 tissue types on 587k H&E WSIs and 195,000 clinical reports. Virchow and PRISM were trained for ×20 magnification, while other variants were trained for ×5, ×10, ×20, and ×40 magnification.

### 5.2 Visiopharm and DeepPath LYDIA

Approximately one in seven women develops breast cancer (4). Breast cancer AI solutions promised enhanced diagnostics by identifying invasive tumors and LN metastases in WSIs (63), with the prospect of quantifying hormonal status, cancer grading, and assessing neoadjuvant chemotherapeutic responses. Mitotic count evaluations have prognostic implications, while TILs are associated with better prognosis. Biomarker phosphorylated Histone H3 was suggested for confirming the presence of mitotic figures. An experimental solution named IMPRESS leveraged H&E and IHC staining, with the IHC-stained images enhancing tumor microenvironment characterization. Noted commercial products included Mindpeak for automating image analysis in human invasive breast carcinoma, OWKIN RlapsRisk™ BC for determining appropriate treatment pathways, and certified Visiopharm products for determining estrogen receptor status (positive or negative). The Visiopharm product could identify invasive tumor regions, as well as detect and measure metastases in H&E breast and rectal adenocarcinoma slides. The Visiopharm Integrator System metastasis AI algorithm demonstrated sensitivity and NPV of 100%.

The Visiopharm website (97) describes their CE-IVD-certified metastasis detection AI (4, 97), reporting improved sensitivity and specificity compared to manual methods (97). It detects metastases in LN H&Es for breast and colorectal carcinoma, measures diameters and areas of metastases, and provides metastasis probability heatmaps. A single-center study at Utrecht assessed the Visiopharm Metastasis Detection app (VMD) on H&E sentinel node (SNs) specimens, also utilizing Visiopharm's viewer app from inside Sectra PACS (4). Costly routine IHC use on sentinel LNs with morphologically negative slides could be reduced by using an approach with “excellent sensitivity”, thereby limiting FN risk (27). Morbidity could be reduced by targeting only axillary SNs for removal (4).

Between unassisted and AI-assisted pathologists, improvements included sensitivity, NPV, time efficiency, detection of smaller metastases, and cost savings by reducing the need for IHC. The study accounted for the metastasis size differences by using a log-binomial model for adjusted relative risk, finding lower adjusted relative risk of IHC use with the AI-assisted workflow. Standalone AI performance demonstrated 95.8% sensitivity, missing one micro-metastasis while detecting all macro-metastases, with a sensitivity of 44.4% for ITCs. International guidelines consider ITCs the markers of residual disease only in patients having undergone neoadjuvant therapy. Following this trial, Utrecht replaced routine IHC metastasis detection in breast sentinel LNs with the VMD (27), with IHC confined to assessing neoadjuvant treatment response cases following a negative result. A confounding factor was a heavily cauterized (4) region on an H&E slide.

VMD was contrasted with CE-IVD-approved (27) DeepPath LYDIA (DPL) (98) in within-distribution and out-of-distribution contexts across six tumor types in a retrospective study of WSIs from 455 patients (27). VMD was approved for colorectal and breast cancer LN assessment. DPL was approved for colorectal, breast, lung, and melanoma LN metastases. Both products were considered excellent for macro-metastases, with DPL more sensitive for micro-metastases and ITCs, particularly in lung cancer and melanoma. VMD generated more FPs. Both exhibited 100% sensitivity within distribution for colorectal cancer and breast cancer macro-metastases, dropping to 95.8% for micro-metastases and 44.4% for breast cancer ITCs. They exhibited 100% sensitivity on out-of-distribution vulvar cancer, and DPL exhibited 100% out-of-distribution sensitivity in head and neck cancer, demonstrating that AI can even operate better outside of intended use. While these AI could flag FNs produced by the pathologists at the slide level, the outcome was inconsequential at the patient level, which illustrates the benefit of assessing multiple slides per patient.

### 5.3 Ibex and Galen products

Ibex provides prostate, breast, and gastric cancer solutions (39), holding CE-IVD clearances, UK MHRA registration, and research-only use in the USA (99). Ibex Galen Breast AI aimed to enhance throughput by detecting invasive cancer and DCIS to provide accurate diagnosis and treatment planning (63). It provided case prioritization, heatmaps, IHC preorders, grading, and AI-driven reporting. Galen Breast produced invasion, ADH, and DCIS scores per slide (1). Invasive carcinoma detection demonstrated AUC 0.99, specificity 93.57%, and sensitivity 95.51%, with subtyping (21). For DCIS, AUC 0.98, specificity 93.79%, and sensitivity 93.20% were reported, along with a grading capacity. Stromal TIL identification demonstrated AUC 0.965. The Welsh Government trialed Ibex Galen AI for breast cancer following successful AI trials with prostate biopsies (29).

A multi-reader study assessed the Ibex Breast HER2 system (37) using on IHC WSIs from four international laboratories. Each site used its own antibody staining protocols. The objective was to identify HER2-low cases, with HER2 0, HER2 1+, HER2 2+, and HER2 3+ scoring categories important for determining therapy. AI-assisted scoring was compared with manual digital scoring, with ground truth determined by five international experts, who themselves had 72.4% interobserver agreement. Interobserver agreement between pathologists increased from 75% to 83.7% with AI use, with HER2 scoring improving from 85.3% to 88.0%. Interobserver agreement for HER2 0 discrimination from 1+ was significantly higher, although accuracy increase from 81.9% to 88.8% was not statistically significant. Standalone AI accuracy across all categories was 92.1%. Cell detection and classification were performed by two separate CNNs. Computational steps included (1) tissue and invasive tumor region detection, (2) individual tumor cell detection within those regions, (3) classification according to IHC membrane staining intensity and completeness, and (4) HER2 scoring.

Ibex Prostate Detect (formerly GSR) analyzed H&E WSI from prostate core needle biopsies as a “safety net” to detect small and rare prostatic cancers (39). Reported PPV cancer heatmap accuracy was 99.6%. Ibex's Galen Prostate AI algorithm gained FDA approval (32), and like Paige Prostate (26) sought detection (96.7% specificity, 96.6% sensitivity, AUC 0.994, NPV 99.2%) and Gleason grading between Grade 1 and higher grades (82.1% specificity, 81.1% sensitivity, AUC 0.901) at the slide level (32). At case level, no cancer cases were missed. The solution alerted for AI versus pathologist diagnosis discrepancies. GSR was integrated into routine frameworks at a CLIA-certified precision pathology laboratory.

In the foundational study (41), training was conducted on ∼1.36M image patches from 549 manually annotated slides, with an internal test dataset of 2,501 slides (cancer detection AUC 0.997, sensitivity 99.6%, specificity 90%, NPV 99.9%) and external dataset of 1,627 slides across 100 cases (cancer detection AUC 0.991, 98% sensitivity, 97% specificity, NPV 99.1%). The AI evaluated cancer probability and percentage, Gleason score, and perineural invasion (AUC 0.957, sensitivity 87%, specificity 91%). Gleason scoring achieved AUC 0.941, sensitivity 86%, and specificity 90% for low-grade (Gleason score 6 or Atypical Small Acinar Proliferation) vs. high-grade (Gleason score 7–10) differentiation using the external dataset. Alert thresholds were set at specificity 90% with corresponding sensitivity ∼99%. Pathologist–AI agreement demonstrated a Pearson correlation of 0.882.

GSR was not intended as a standalone diagnostics solution (50, 100). The system was cloud-hosted and integrated with a scanner and IMS for input “ingestion” to provide ANN-based classification (high vs. low grade). Standalone 510(k) performance evidence showed slide-level sensitivity 81.0% and specificity 91.6%, case-level sensitivity 80.8% and specificity 46.9%, unexpectedly lower for sensitivity at the case level. Human-in-the-loop performance improved sensitivity to 93.9% and specificity to 87.9%—a 3.5% sensitivity improvement on standard of care, but a −3.2% decrease in specificity.

Galen Prostate and Galen Breast were validated retrospectively against a Japanese cohort of 100 cases each with AUC 0.969 and 0.997 for detection, respectively (1). Galen Prostate distinguished between low-grade and high-grade adenocarcinoma with AUC 0.994. Low and medium grades were distinguished with 97.9% sensitivity and 92.9% specificity. The algorithms were originally trained and tested for an Israeli cohort, and consequently the solution was deemed to generalize well across populations. Galen Prostate corrected four Gleason scores and detected one missed cancer, representing a 5% revision rate. The authors suggested that further training on subtypes and benign lesions, such as intraductal papilloma and fibroepithelial tumors, could enhance differentiation.

### 5.4 MSIntuit by Owkin

MSIntuit is a CE-IVD-marked (84) pre-screening AI tool for dMMR and MSI detection from colorectal histology H&E slides, with tile-level (112 × 112 μm) scoring (31). MSI arises from dMMR, representing failure of DNA replication error correction. The MSI biomarker has prognostic and therapeutic importance, with universal screening on new diagnoses recommended by NICE and the National Comprehensive Cancer Network. MSI is found in 15% of colorectal cancer patients and was the first FDA-approved pan-cancer biomarker. Mismatch repair loss is detectable by IHC, polymerase chain reaction, or expensive next-generation sequencing. Using an accelerated AI workflow, MSI-positive patients can be selected for further IHC or PCR tests, thereby providing triage.

MSIntuit aimed to rule out non-MSI patients using H&E colorectal tumor samples via binary classification, with blind validation on an independent dataset of 600 patients (sensitivity 0.96–0.98, specificity 0.46–0.47). Four slides were missed due to good differentiation. Inter-scanner agreement was tested (Cohen's *κ* 0.82), with calibrated decision thresholds numerically similar and tile-level scores correlated (R = 0.92). Training utilized 4 million TCGA-COAD (Colon Adenocarcinoma) tiles, with a 50-layer ResNet50 feature extractor (2,048 features) trained using Momentum Contrast (MoCo) v2 SSL to produce similar representations under heavy data augmentation. This SSL approach was more effective than ImageNet-based approaches and more robust to scanner variations. Following tile-level feature extraction, a Chowder-based MLP scored the tiles, with the top and bottom ten scores concatenated for aggregation by another MLP as a slide-level prediction.

### 5.5 Stratipath breast

Stratipath Breast was the first CE-IVD-marked H&E WSI AI solution for prognostic low- and high-risk group binary stratification for routine clinical use in breast cancer (23). It was validated against two independent breast cancer cohorts, using one WSI per patient. Multivariate CPH, adjusted for covariates, demonstrated hazard ratio (HR) 2.76 for progression-free survival between high- and low-risk groups in an estrogen receptor-positive and HER2-negative (ER+/HER2−) patient subgroup. When Nottingham histological grade 2 was included in this subgrouping, the HR was 2.20. With ER+/HER2− considered the most critical subgroup to stratify therapeutic decision-making, the tool was put forward for decision support. Progression-free survival was defined in terms of “time to local recurrence, distant metastasis or detection of contralateral tumors.”

### 5.6 DeepDx Prostate by Deep Bio Inc.

The CE-marked DeepDx Prostate commercial AI was developed by South Korea-based Deep Bio Inc. (2), a company associated with CGC success (72). Single-center external validation was sought (2). With potential differences in whole-mount radical prostatectomy and biopsy representations, the study reported on generalization to radical prostatectomy. The algorithm demonstrated high agreement with pathologists (Cohen's *κ* 0.91). Gleason pattern localization, speed, and efficiency were deemed superior to human pathologists. Gleason patterns were quantified by segmentation. Gleason grading ground truth was determined at tile level by a consensus of two pathologists, with a third serving as tiebreaker. Agreement between two expert pathologists and this reference yielded a quadratically weighted Cohen's *κ* 0.94 (unweighted 0.73) vs. the AI's 0.89 (unweighted 0.56). For cancer vs. non-cancer classification, an unweighted Cohen's *κ* of 0.91 was determined for the AI against reference (sensitivity 0.997, specificity 0.88, NPV 0.99, PPV 0.95).

### 5.7 DoMore! project

Histotype Px Colorectal (previously DoMore-V1-CRC) was a CE-IVD product associated with the DoMore! Project (34), trained and tested on 4,500 patient samples and 90 million image tiles, with multi-site slide preparation using two different scanners. Slides were partitioned into non-overlapping tiles (69, 70). Two five-model ensembles at ×10 and ×40 magnifications provided probabilities of poor prognosis. The models within each ensemble differed from each other by random initialization and sampling. Decision thresholds were referred to as markers. Consensus between the two ensembles was accepted, while disagreement was deemed uncertain (69–71).

The DoMore-V1-CRC marker was developed using DL on H&E stained sections (35). Risk categories included >90% 3-year survival for low risk, <70% 3-year survival for high risk, and an intermediate class. AI-predicted risk classes aligned with CPH HR expectations, for example, 10.71 between high- and low-risk groups. The system aimed to augment risk stratification by combining existing prognostic markers with AI output to improve adjuvant chemotherapy selection. It was concluded that the new system predicted cancer-specific survival better than current guidelines.

Ten CNNs were developed using ∼12M image tiles from four cohorts for prognostic WSI classification (71). The MIL-based DoMore v1 network was used, with trainable parameters across a MobileNetV2 CNN representation network, a Noisy-AND pooling function, and a fully connected classifier head. The work aimed to stratify stage II and III patient groups to guide the decision of whether to undergo adjuvant treatment, with the stage determining the therapeutic plan. Validation data were sourced from 170 hospitals across 7 countries. Training was conducted using TensorFlow 1.10, with c-index used to select five models at each resolution. Non-overlapping tiles were used (∼11.6M at ×40, ∼635k at ×10). Ground truth was cancer-specific survival, with five models averaged or voting for the probability of poor prognosis. Thresholds were set according to tuning cohort evaluations. HR was 3.04 between poor and good prognosis after adjustment for established markers (pN and pT stages, lymphatic invasion, venous vascular invasion). The AI assay outperformed established molecular and morphological prognostic markers for 3-year survival classification of good versus uncertain or poor prognosis (sensitivity 52%, specificity 78%, PPV 19%, NPV 94%, accuracy 76%).

### 5.8 Artera Prostate

ArteraAI Prostate was FDA-approved at risk class II on 31 July 2025, for use alongside an FDA- or 510(k)-cleared scanner (62), to provide prognostic risk decision support based on treatment-naïve prostate core needle biopsy H&E WSIs. Two multimodal algorithms from Artera Prostate Prognostic Model version 1.1 were previously developed using histopathology images and clinical variables (80). These algorithms, trained using MIL with attention on data from five phase III trials, had outperformed standard risk models. External validation was conducted on 318 men from a phase III randomized trial involving androgen suppression and radiotherapy with or without adjuvant combination therapy. The two trial arms showed no statistical difference in endpoints of interest and were pooled together. Primary endpoints were time to distant metastasis and prostate cancer-specific mortality. Secondary endpoints were biochemical failure, death with metastasis, and overall survival (time to death). The aim was risk stratification in high-risk or locally advanced prostate cancer. Effective stratification was needed, since indolent cases risked overtreatment, while high-risk cases risked undertreatment. The two algorithms demonstrated prognostic separation (Fine-Gray sHRs of 2.33 and 3.54 for respective endpoints). CPH regression was used for overall survival. Gleason grade, prostate-specific antigen, and T stage demonstrated poor to modest prognostic capacity, while loss of glandular structure and tissue organization were associated with disease trajectory. A stated study limitation was the lack of genomic biomarkers or clinical nomogram comparators.

### 5.9 Genius by Hologic

The Genius™ Digital Diagnostics System with the Genius™ Cervical AI algorithm by Hologic Inc was developed for expert cytologist evaluations of ThinPrep Pap Test glass slides for cervical screening (50, 101). It was engineered to assess Bethesda System cytological categories, including atypical cells, neoplasia, precursor lesions, and carcinoma. Ambiguities were reassessed by light microscopy. With CE-IVD (2021) and FDA (2024) approval, the solution was independently validated (51) using 890 reviewed ThinPrep Pap tests. The study noted routine use in Europe, aiming to evidence performance at a US institute. Sensitivity 98.2%–100% was determined for classification of atypical squamous cells of undetermined significance or higher (92.4%–100% NPV). A minimum consensus of two out of three pathologists using the system was considered concordance (Kendall *W* coefficient 0.909). Discordance was found in 11 out of 890 cases, which were re-reviewed. The solution used a volumetric stack of merged images, with a digital gallery highlighting diagnostically relevant images and objects. Review time improved for pathologists and cytologists as they acclimatized to the system. Review times per slide were 37s–77s compared to 3 to 5 min for a Hologic light microscope solution.

### 5.10. Lunit SCOPE PD-L1

The CE-IVDD-marked Lunit SCOPE PD-L1 is a PD-L1 biomarker TPS analyzer, trained on >1,000,000 cancer cell images (102) and promoted for “broad cancer types” (103). A Lunit AI study identified PD-L1 presence as pivotal to immune checkpoint inhibitor therapeutic response (36). Training used 393,565 tumor cells annotated as PD-L1-negative or PD-L1-positive by 33 board-certified pathologists across 4,675 IHC stained tissue grids from 802 WSIs, with validation on 430 external WSIs. External IHC slides were annotated by three pathologists with consensus taken as mean PD-L1 TPS. TPS was classified into <1%, 1%–49%, and ≥50%, with concordance of 85.7%, 89.3%, and 52.4%, respectively. The AI counted PD-L1-positive and PD-L1-negative tumor cells on grids with ≥20 tumor cells. Agreement between AI and pathologists was demonstrated with Spearman coefficient (0.925). Using the lowest-risk group as reference (TPS ≥ 50%), AI stratification for TPS demonstrated higher HRs for <1% and 1%–49% groups than pathologist stratification. Processing was performed via OpenSlide, and the cell detection pipeline utilized a “Faster Region-Based” CNN with ResNet-101 feature extractor. Tumor cells were marked with dots and bounding boxes. Soft Dice loss addressed class imbalance for object classification loss. Global Average Pooling was followed by a fully connected layer for classification and regression, and hyperparameter tuning was used. TPS classifications were contrasted against objective response rate, progression-free survival (HRs), and overall survival, enabling comparison of AI and pathologist prognostic performances.

## 6 Research-only examples

An increasing number of proprietary software solutions have gained international certification for medical applications derived from open-source software (25). Furthermore, *de novo* development of pathology workflows has been discouraged for reasons of efficiency and reproducibility. This section highlights selected state-of-the-art research to consider for future AI pipelines.

### 6.1 STAMP

STAMP (25) was an open-source proof-of-concept DL approach for biomarker prediction from H&E WSIs. STAMP represented a standardized H&E preprocessing pipeline (22), reported when coupling a pretrained feature extractor (cTransPath) to a Transformer architecture for weakly supervised classification (25), although it was highly modular (104). It extracted features from non-overlapping patches, producing 768 dimensions per 150,528-dimensional patch (224 × 224, with three color channels) (25). The features were passed to a Visual Transformer (ViT) model (two layers, eight attention heads) with its patch embedding layer removed. Early stopping was monitored over 16 epochs by AUROC against validation data, and data augmentation was excluded. The model assigned each patch an importance score. AUROC 0.85 and AUPRC 0.68 were reported on external data, and CPH was conducted for prognostic assessment. “Standards for Reporting of Diagnostic Accuracy Studies” and “Transparent Reporting of a multivariable prediction model for Individual Prognosis or Diagnosis” were proposed to evaluate clinical utility. STAMP was available as a complete modular pipeline, including for slide-level encoding using TITAN (104).

### 6.2 TITAN

TITAN represented a higher-level multimodal FM ViT for WSIs, stacked downstream upon patch encoder FM outputs (10), that is, a slide-level encoder. It incorporated Euclidean 2D adapted ALiBi-based positional encoding. TITAN was evaluated for few-shot learning generalizability and pretrained on 335,645 WSIs across 20 organs and 182,862 medical reports. ALiBi positional encoding was effective and was operated by adding a bias to the Q-K dot product to penalize tokens at a distance when computing the attention score. TITAN was trained across 89.7% H&E, 7.9% IHC, 2.3% special stains, and 0.1% other types of slides. The dataset was diverse across scanners. TITAN-visual was a 12-headed, 6-layered Transformer-based encoder trained using SSL from CONCH patch-level encodings, with masked modeling of patch embeddings and knowledge distillation using the iBOT (105) framework. CoCa vision-language modeling used pathology reports, and Qwen2-7B-Instruct rephrased synthetic captions generated by PathChat upon prompting on patches for morphological descriptions (10). TITAN could generate pathology reports; however, the multimodal vision-language decoder was withheld due to the risk of leaking protected data. TITAN was compared to PRISM, GigaPath, and CHIEF, and consistently outperformed mean pooling and ABMIL using the same patch encoder.

### 6.3 Pathchat, Paige Alba, and multimodal, generative, agentic AI

While LLMs and multimodal agentic models had not achieved regulatory approval in healthcare (9), PathChat was a multimodal generative ChatGPT-like AI “copilot” trained on image–text instruction pairs (20). PathChat earned FDA Breakthrough Device Designation (54). Paige Alba also offered a copilot experience (26) and was integrated with FMs, with multimodality across pathology, radiology, and clinical data. It supported voice and text commands, permitting report consolidation and generation, order placements, cancer subtype detection, genomic biomarker prediction, zero-shot detection, and visualization of suspicious areas. Integration with OmniScreen allowed for genomic alteration predictions across 491 genes in multiple cancers, using molecular gene panels. Agentic multimodal tools like Paige Alba may well be “the future of precision medicine through integrated diagnostics.”

## 7 Discussion and conclusions

Our objective to provide an informative assessment of the regulatory and translational climate in AI digital WSI histopathology was achieved. This review highlighted global regulatory-approved AI histopathology implementations (Table 4), while also discussing a small cross-section of state-of-the-art research-only examples. A wide range of technologies, statistical techniques, and commercial validation studies were reviewed. Interestingly, World Health Organization (WHO) involvement was not present within our AI-related investigations despite using a wide range of contemporary sources (Table 2). Identifying information on H&E AI products is a known challenge (30), and concerns related to international collaboration and dataflows have been raised (13). Moreover, disease classification guidelines potentially vary between countries (33). Thus, improved standardization and interpretation of non-computational clinical decision algorithms, including those by the WHO (106) and the International Consensus Classification (107), may help identify clinically relevant ML targets through pathologist-informed consensus (6, 25). Critical for AI training and validation is the production of high-quality, diverse datasets, including rare conditions and across modalities.

As noted in Section 2, the FDA provided substantially more robust search capacity than either MHRA or EUDAMED, with FDA search capabilities explicitly highlighting emerging AI technologies. Consequently, this review was better able to characterize the FDA space than UK pathways, European pathways, or specific laboratory exemptions. For instance, searches for Paige on EUDAMED and MHRA yielded basic use case, market registration information, and risk classification, but involved difficult-to-navigate categories that do not directly identify AI devices and lacked validation evidence. By contrast, the FDA explicitly isolates AI tools with full FDA approval reports, including validation details (49) and dataset demographic characteristics, which can facilitate recognition of device bias. For instance, research-use ML for mature B-cell neoplasm subtyping reported Chinese vs. Western population differences in mutation patterns (108). Consequently, geographic, demographic, and socioeconomic dataset composition warrants care. A further limitation of emphasizing regulatory-approved solutions is the reliance on validation studies for proprietary, closed-source solutions.

Hyperparameter optimization (36) and architectural experimentation with variable performance (10) were exhibited, but further automation of neural architecture search may warrant investigation. Increasing use of state-of-the-art architectures, including transformer models (10, 22, 25), multimodal approaches, and more generalizable pattern recognition systems, may be expected, with reduced reliance on traditional CNN models. However, regulatory-approved agentic and generative AI applications remain a major research hurdle (26, 54), given the safety, accountability, and validation challenges posed by increasingly autonomous clinical systems, including concerns about patient data reconstruction from generative models (10). Notably, there were no explicit regulatory concerns regarding generative coding; rather, further automation of code validation was sought (13).

Pan-cancer generalization is sought in state-of-the-art research (10). Promising pan-cancer generalization was demonstrated with VMD and DPL for LN metastasis detection across tumor types, although with practicality restricted by high FP rates (27). Of note was an emphasis on sensitivity over specificity by threshold selection (31, 32, 41, 48), narrowing the search space for human pathologists while minimizing AI-related risks. Furthermore, AI-guided insights may enhance human pattern recognition and decision-making, assessed through quantifiable reductions in interobserver variation (36–38). Visual AI model explainability may support future pathologist training, where enhanced segmentation models (2, 31, 72, 81) may improve local WSI interpretability and biomarker quantification, with a corresponding need to reconcile the FP alert problem (4, 27, 32) and confounding histological characteristics (41), which can influence model reliability in identifying foci.

With well-defined objective functions, pattern recognition algorithms can guide precision therapeutic interventions toward biomarker targets (23, 31, 35–37), although challenges related to establishing therapeutic protocols remain across disease and senescence (109). While survival analysis and statistical prognostic outcomes are important targets, considering tissue-specific indicators such as biomarkers, histological architecture, cytology, and omics may be a more direct path to regenerative strategies. This may include more explicit parametric comparison with healthy control states. A study related to H&E AI products identified that 18/26 involved detection, grading, and quantification, 3/26 involved prognosis and treatment, and 5/26 involved biomarker prediction (30).

The scope of AI histopathology warrants expansion. For example, in alignment with our group's hematopathological collaboration, establishing regulatory pathways for diagnostic lymphoma AI via the FDA and European Medicines Agency was considered “critical” (33). Although lymph node metastasis AI was identified (27, 63, 97, 98), our investigation did not reveal lymphoma-specific regulatory-approved AI, nor were such solutions detailed within contemporary lymphoma AI reviews (19, 33). A search of the EUDAMED database for “Device types: Software,” “Medical purpose of the system or procedure pack: lymphoma,” and “Status: On the EU market” returned “No records found.” Nevertheless, AI research in lymphoma is well represented (19), including the capacity to quantify features such as lymphocyte aggregation patterns, although with emphasis on B-cell lymphoma, with less representation for T-cell lymphomas, Hodgkin lymphoma, and other rare subtypes (19). This was a concern given the aggressiveness and heterogeneity of these subtypes. LymphoML was a solution for classifying multiple lymphoma subtypes from H&E (33). Furthermore, a multimodal relapse model was described integrating clinical, pathological, and molecular data at AUC 0.87.

Histopathology via H&E and immunophenotyping remains the gold standard in lymphoma diagnosis (19). By contrast, PET and CT are gold standards in lymphoma staging, treatment response, and prognostication (9), with AI promising enhanced tractability in quantification for prognosis and treatment response, for instance, in estimating total metabolic tumor volume and total lesion glycolysis using CNNs. Alternatively, fluorescence *in situ* hybridization is also commonly deployed in high-grade B-cell lymphomas (19), as well as molecular testing for genetic aberrations (33). Together, these modalities highlight the potential importance of direct multimodal integration in relevant contexts beyond merely SSL-based representational enhancement (10, 18, 22, 31).

Among the commercial systems reviewed, explicit FM use was already evident within the Paige ecosystem (26). Specialized FMs may increasingly be adopted beyond WSI analysis, presenting comparable regulatory validation challenges. This is highlighted by vision-language model development such as CT-CLIP (110) for 3D CT-report alignment via contrastive learning, MAIRA-2 (111) for spatially localizing radiological findings within chest X-rays alongside automated report generation, and BiomedCLIP (112) for cross-modal representation learning across 15 million biomedical image–text pairs spanning histology, radiology, and microscopy.

AI has wider applicability than histopathology, including in cytology (20). In radiology, Limbus Contour is on the European market (113), providing DL-based segmentation for radiation therapy, reported as 5–10× faster than manual contouring (114). CE-marked radiology products were available via the Health AI Register (30, 115). The Royal College of Radiologists and NHS England also developed a register of in-use regulatory-approved AI products (30, 116). Multi-omics integration with histopathological images for multimodal implementations was noted for future precision oncological treatments (19), with genotypic and expression biomarkers increasingly matched to anticancer therapy (71). Single-cell RNA and DNA sequencing can provide high-resolution cellular profiles within tumors (33). While spatial technologies provide high-resolution data across a vast spectrum of biomarkers, such solutions tend to be implemented as “For Research Use Only” (117, 118), as also discerned from the Festival of Genomics and Biodata 2026 in London and Spatial Biology 2026 in Newcastle, where economic challenges to clinical adoption were highlighted. Nevertheless, spatial biology assays represent a likely avenue through which crucial disease-associated biomarkers can be discovered and may be useful for regenerative therapy through organoid evaluation (119).

Evidently, resolving challenges across dataset availability and data acquisition, problem formulation for clinical relevance, domain-specific expertise across commerce and technology, computational resource access and training, appropriate external and independent validation, completion of clinical trials, pathologist acceptance, and cost and logistical issues associated with modality enhancement, such as IHC and spatial profiling technologies, could accelerate AI adoption, improve effectiveness, and broaden applicability to rare diseases, finer-grained subtyping, and stratification.
