# Supplementary material for: Translational AI in whole-slide image cancer histopathology: state of the art and regulatory-approved solutions
Source: Front Digit Health. 2026 Jul 9;8:1863382. doi: 10.3389/fdgth.2026.1863382 (PMC13391564; doi:10.3389/fdgth.2026.1863382)
Supplement: Supplementary file 1 [file Datasheet1.pdf]

Supplementary Material

1 Expert Supervision, Multidisciplinary Collaboration, and Documentation

Appropriate use of multidisciplinary [1] expertise and tooling was advised [2] for AI integration into clinical workflows. Business expertise may also be beneficial [2]. Many AI use-cases were considered ill-conceived [3], with biologists better equipped to devise problem statements, handle data curation, and interpret clinical relevance [4]. Engineers should prioritize data manipulation for generalizable outcomes.

Fully up-to-date documentation, including development pipelines and learning mechanisms, can accelerate market approval [5], as illustrated by the Artera Prostate clearance report [6]. Clear user instructions for healthcare professionals and/or patients [1] with open communication channels [1, 2] facilitate correct device operation. The FDA noted that transparency can more than double device adoption [5], while the UK government promoted the transparency offered by open-source models, lawfulness, public awareness, and close collaboration between commercial and governmental partners [2].

2. Hardware Requirements

Computational demands vary by application. A GPU with at least 40 GB memory was recommended for training Transformer architecture, with at least 10 GB for preprocessing [4]. TITAN visual was trained using four NVIDIA A100 80GB GPUs [7]. Vision-Language training used eight NVIDIA A100 80GB GPUs. However, following pretraining further experiments on TITAN were performed on a single NVIDIA 3090. Experimentation on pretrained patch encoders used 40 GB NVIDIA RTX A6000 and L40 GPU nodes. A validation study of the clinically deployed Galen Prostate solution by Ibex used four GPUs running parallel to pathologists, complete with WSI scanner and server [8]. QPMIL-VL experimentation used two dual RTX 3090s [9]. The learnable parameter size was a modest 0.365M. A DoMore! Project solution described median 2.8 mins processing time per patient from image to prediction on a NVIDIA RTX 2080 Ti and Intel Core i7-7700K [10].

3. Concatenation for Multiscale or Multimodality

Multiscale approaches used feature concatenation across multiple resolutions [11]. Alternatively, a study [12] trained models for different resolutions, with outputs combined through attention-pooling [11]. Concatenation was also used in feature fusion to train a prostate cancer risk classifier, conducted between image representations, clinical data, National Cancer Centre Network variables, and Gleason characteristics [13]; based upon which ArteraAI submitted for FDA approval.

4. Incremental Learning and QPMIL

Patch-level encodings were extracted using CONCH [9], and a set of learnable prototype keys was updated incrementally across datasets for use in a prototype-query mechanism. Max pooling was applied to patch features to create a "query vector" for comparison against prototype keys via cosine similarity, with the top N prototypes selected and a penalty on overused prototypes to avoid catastrophic forgetting.

The selected prototypes were fed to the text encoder, with outputs compared to each patch-level embedding, resulting in a cosine similarity matrix. SoftMax was applied per prototype to derive a weight matrix for multiplication against the original patch embeddings, which were averaged for a single vector representation of the WSI. For classification, multiple textual paraphrases of a class were encoded by Conch text encoder and averaged, with a learnable vector added to produce "enhanced class features". These were compared against the WSI embedding via cosine similarity to determine the predicted class.

Incremental learning was evaluated forwards and backwards across four sequential datasets, achieving an accuracy of 0.890, reported as SOTA in incremental learning. A performance drop of 0.049 under reversed ordering was attributed to the largest dataset inducing partial forgetting when put last. Post hoc clustering of learned prototype features demonstrated clear dataset separation, alongside shared prototypes.

5. TCGA Acquisition Method

TCGA and other datasets can be found via the National Cancer Institute Genomic Data Commons Data Portal [14]. From the TCGA project pages manifest files are available, which are a list of data available for the specific project. Although applicable to other datasets, we provide a simple illustrative method we used to acquire TCGA-BRCA [15] WSI slides. Our method used Windows but should be adaptable to other operating systems. The GDC Data Transfer Tool requires download [16]. This client application would reside in the same folder as a manifest. The manifest was filtered for only SVS files containing the WSI and by sample types. SVS files are commonly used for WSI AI, including in regulatory approved validation trajectories [17]. Other formats include BIF, TIF, and TIFF [13]. Tumor types could be identified via TCGA Barcode [18]. From the barcode we could identify unique patient IDs, the number of metastatic classified slides, the number of solid tumor slides, and the number of normal classified slides. Subsequently a .bat file was written as follows to download specific SVS:

```
@echo off
E:
cd "E:\TCGA-BRCA\ "
gdc-client.exe download -m "E:\TCGA-BRCA\manifest_primary_solid_tumor_ILC-lobular.tsv" --
debug
pause
```

In this case, *manifest_primary_solid_tumor_ILC-lobular.tsv* represented a manifest containing only Invasive Lobular Carcinoma SVS slides.

Classifications were parsed from corresponding patient XML files also available via manifest. However, classifications and other data may be more readily retrieved via the corresponding "Clinical" download option on the project page [15]. Once downloaded, SVS format WSIs were visualized and manipulated with QuPath-0.6.0.

6. Imputation

Missing clinical variables were imputed using sklearn KNNImputer [19]. Imputation introduces inferred values, with biasing risk or informational leakage if using an inappropriate imputation

model. However, KNN may be justified as a feature-space-based value estimation method, and rigorous external validation may mitigate imputation risks.

7. Language Model Evaluation

Report generation was evaluated using METEOR, ROUGE, or BLEU [7] with analytics via linear mixed-effects modelling and two-sided Wald z tests on the fitted model. BLEU was used in modelling TITAN for histopathology, and also in the seminal Transformer paper [20]. GOV.UK Chat contrasted AI outputs to human via the Likert scale, with no significant differences found [2]. Cross-modal retrieval can be measured with Recall@K, while slide retrieval can use Acc@K and MVAcc@5 [7].

8. Confidence Intervals

AI uncertainty can be measured, and the FDA considered confidence interval (CI) reporting essential [5]. Variance assessments, such as standard deviation and percent coefficient of variation, provide insights regarding reproducibility [5]. Bootstrap resampling can be conducted on a final model for error bars and CIs [4, 7], for example bootstrapping with 1000 iterations [21, 22]. In an Ibex validation study, 95% CIs for sensitivity and specificity were determined using bootstrapping [23], and AUC CIs were determined by the Wald method [8, 23]. In the DoMore! Project, bootstrapping was used to determine CIs for net reclassification improvement, c-indices, and AUCs, with acceleration constant estimated via leave-one-out cross-validation [10]. CIs can be estimated via cross-validation ($CV = \frac{1}{k}\sum_{i=1}^{k} val\_score_i$), with each fold (k) contributing to the performance distribution [7]. Thus, "fold instability" can be used to determine standard deviations [9, 24]. Another method used the exact binomial method to calculate 95% CIs for sensitivity and NPVs [25].

References

1. FDA, H.C., MHRA. *Good Machine Learning Practice for Medical Device Development: Guiding Principles*. 2021 [cited 2025; Available from: https://www.fda.gov/media/153486/download.

2. Government_Digital_Service. *AI Playbook for the UK Government*. 2025 [cited 2025; Available from: https://www.gov.uk/government/publications/ai-playbook-for-the-uk-government.

3. Sokol, K., J. Fackler, and J.E. Vogt, *Artificial intelligence should genuinely support clinical reasoning and decision making to bridge the translational gap.* npj Digital Medicine, 2025. **8**(1): p. 345.

4. El Nahhas, O.S.M., et al., *From whole-slide image to biomarker prediction: end-to-end weakly supervised deep learning in computational pathology.* Nature Protocols, 2025. **20**(1): p. 293–316.

5. FDA. *Artificial Intelligence-Enabled Device Software Functions: Lifecycle Management and Marketing Submission Recommendations*. 2025 [cited 2025; Available from: https://www.fda.gov/regulatory-information/search-fda-guidance-documents/artificial-intelligence-enabled-device-software-functions-lifecycle-management-and-marketing.

6. Administration, U.S.F.a.D. *Device Classification Under Section 513(f)(2)(De Novo) (ArteraAI Prostate)*. 2025 [cited 2026; Available from: https://www.accessdata.fda.gov/scripts/cdrh/cfdocs/cfpmn/denovo.cfm?id=DEN240068.

7. Ding, T., et al., *A multimodal whole-slide foundation model for pathology.* Nature Medicine, 2025.

8. Pantanowitz, L., et al., *An artificial intelligence algorithm for prostate cancer diagnosis in whole slide images of core needle biopsies: a blinded clinical validation and deployment study.* Lancet Digit Health, 2020. **2**(8): p. e407–e416.

9. Gou, J., L. Ji, P. Liu, and M. Ye, *Queryable Prototype Multiple Instance Learning with Vision-Language Models for Incremental Whole Slide Image Classification.* Proceedings of the AAAI Conference on Artificial Intelligence, 2025. **39**(3): p. 3158–3166.

10. Skrede, O.-J., et al., *Deep learning for prediction of colorectal cancer outcome: a discovery and validation study.* The Lancet, 2020. **395**(10221): p. 350–360.

11. Gadermayr, M. and M. Tschuchnig, *Multiple instance learning for digital pathology: A review of the state-of-the-art, limitations & future potential.* Comput Med Imaging Graph, 2024. **112**: p. 102337.

12. Hashimoto, N., et al. *Multi-scale Domain-adversarial Multiple-instance CNN for Cancer Subtype Classification with Unannotated Histopathological Images*. in *2020 IEEE/CVF Conference on Computer Vision and Pattern Recognition (CVPR)*. 2020.

13. Zhang, D.Y., et al., *Implementation of Digital Pathology and Artificial Intelligence in Routine Pathology Practice.* Laboratory Investigation, 2024. **104**(9).

14. National_Cancer_Institute. *Genomic Data Commons Data Portal*. 2025 [cited 2025; Available from: https://portal.gdc.cancer.gov/.

15. National_Cancer_Institute. *Project TCGA-BRCA*. 2025 [cited 2025; Available from: https://portal.gdc.cancer.gov/projects/TCGA-BRCA.

16. National_Cancer_Institute. *GDC Data Transfer Tool*. 2025 [cited 2025; Available from: https://gdc.cancer.gov/access-data/gdc-data-transfer-tool.

17. da Silva, L.M., et al., *Independent real-world application of a clinical-grade automated prostate cancer detection system.* The Journal of Pathology, 2021. **254**(2): p. 147–158.

18. National_Cancer_Institute. *TCGA Barcode*. 2025 [cited 2025; Available from: https://docs.gdc.cancer.gov/Encyclopedia/pages/TCGA_Barcode/.

19. Ross, A.E., et al., *External Validation of a Digital Pathology-based Multimodal Artificial Intelligence Architecture in the NRG/RTOG 9902 Phase 3 Trial.* European Urology Oncology, 2024. **7**(5): p. 1024–1033.

20. Vaswani, A., et al., *Attention Is All You Need.* arXiv [cs.CL], 2023.

21. Flach, R.N., et al., *Head-to-Head Comparison of 2 Artificial Intelligence Tools for Detecting Lymph Node Metastases in Whole-Slide Pathology Images Within and Beyond Their Intended Use.* Modern Pathology, 2025. **38**(12): p. 100905.

22. Saillard, C., et al., *Validation of MSIntuit as an AI-based pre-screening tool for MSI detection from colorectal cancer histology slides.* Nature Communications, 2023. **14**(1): p. 6695.

23. Lami, K., et al., *Validation of prostate and breast cancer detection artificial intelligence algorithms for accurate histopathological diagnosis and grading: a retrospective study with a Japanese cohort.* Pathology, 2024. **56**(5): p. 633–642.

24. Dehaene, O., A. Camara, O. Moindrot, A. de Lavergne, and P. Courtiol, *Self-Supervision Closes the Gap Between Weak and Strong Supervision in Histology.* arXiv [eess.IV], 2020.

25. van Dooijeweert, C., et al., *Clinical implementation of artificial-intelligence-assisted detection of breast cancer metastases in sentinel lymph nodes: the CONFIDENT-B single-center, non-randomized clinical trial.* Nature Cancer, 2024. **5**(8): p. 1195–1205.
